# Comprehensive characterization of multi-omics landscapes between gut microbial metabolites and the druggable genome in sepsis

**DOI:** 10.3389/fimmu.2025.1597676

**Published:** 2025-07-21

**Authors:** Jun Liu, Tong Li, Li Xin, Xingyu Li, Jianbo Zhang, Peng Zhu

**Affiliations:** ^1^ Department of Gastrointestinal Surgery, The Second Affiliated Hospital of Chongqing Medical University, Chongqing, China; ^2^ Department of Cardiology, The Second Affiliated Hospital of Chongqing Medical University, Chongqing, China

**Keywords:** GPCRs, ion channels, kinases, Mendelian randomization, microbial metabolites, sepsis

## Abstract

**Background:**

Sepsis is a life-threatening condition with limited therapeutic options. Emerging evidence implicates gut microbial metabolites in modulating host immunity, but the specific interactions between these metabolites and host druggable targets remain poorly understood.

**Methods:**

We utilized a systems biology framework integrating genetic analyses, multi-omics profiling, and structure-based virtual screening to systematically map the interaction landscape between human gut microbial metabolites and druggable G-protein-coupled receptors (GPCRs), ion channels (ICs), and kinases (termed the “GIKome”) in sepsis. Key findings were validated by molecular dynamics (MD) simulation, microscale thermophoresis (MST), and functional assays in a murine cecal ligation and puncture (CLP) model of sepsis.

**Results:**

We evaluated 190,950 metabolite-protein interactions, linking 114 sepsis-related GIK targets to 335 gut microbial metabolites, and prioritized indole-3-lactic acid (ILA), a metabolite enriched in *Akkermansia muciniphila*, as a promising therapeutic candidate. MD simulation and MST further revealed that ILA binds stably to PFKFB2, a pivotal kinase in regulating glycolytic flux and immune activation during sepsis. *In vivo*, ILA administration improved survival, attenuated cytokine storm, and mitigated multi-organ injury in CLP-induced septic mice.

**Conclusions:**

This systems-level investigation unveils previously unrecognized therapeutic targets, offering a blueprint for microbiota-based precision interventions in critical care medicine.

## 1 Introduction

Sepsis is characterized by a dysregulated host response to infection that precipitates life-threatening organ dysfunction (1). Data from the Global Burden of Disease (GBD) 2017 study estimate that sepsis accounts for 19.7% of global deaths, underscoring its profound public health impact (2). Despite advances in antimicrobials, supportive care, and early diagnostics, outcomes remain poor, particularly in cases of multiple organ dysfunction syndrome (MODS). The repeated failure of sepsis trials underscores the urgent need for novel therapeutic strategies, demanding a fundamental shift in our approach to its pathophysiology and treatment (3). Drugs modulate cellular functions by interacting with biomolecular targets, enabling disease treatment and quality-of-life enhancement. Among druggable targets, G-protein-coupled receptors (GPCRs), ion channels (ICs), and kinases (collectively termed the “GIKome” in this study), are among the most frequently targeted protein families, prioritized by the Illuminating the Druggable Genome (IDG) initiative (4). Approximately 800 GPCRs are encoded in the human genome, enabling the detection of a broad spectrum of external signals essential for regulating key physiological functions, including hormonal signaling and neural communication (12). Characterized by a conserved seven-transmembrane helical architecture, they initiate intracellular signaling primarily through interactions with G proteins and arrestins. GPCRs are believed to share a conserved evolutionary lineage and collectively participate in complex signaling networks that regulate numerous physiological and pathological functions. Their extensive biological relevance, coupled with their accessibility to pharmacological modulation, has made them a prime focus in drug discovery, with more than 400 approved therapeutics targeting this receptor family (13, 14). ICs are integral membrane proteins that selectively mediate the flow of key ions such as calcium (Ca^2+^), potassium (K^+^), sodium (Na^+^), and chloride (Cl^-^), thereby maintaining membrane potential and regulating vital cellular processes (15). They are broadly categorized into voltage-gated, ligand-gated, and mechanically activated channels based on their activation mechanisms (16). Beyond their roles in excitability and homeostasis, ICs are critically involved in intracellular signaling pathways linked to oxidative stress and inflammation, two hallmarks of many pathological conditions (17). For instance, by regulating intracellular ion concentrations, especially Ca^2+^, ICs critically modulate signaling pathways that control gene expression, cellular differentiation, and apoptosis (18). Owing to their central physiological roles and sensitivity to environmental stimuli, ICs have emerged as attractive therapeutic targets across diverse pathological contexts. Protein kinases are enzymes that mediate the transfer of phosphate groups from ATP to specific substrates, thereby regulating a wide range of cellular processes through phosphorylation-dependent signaling (19). Based on substrate specificity, kinases are broadly classified into protein, lipid, carbohydrate, and other specialized kinase families, with protein kinases forming the most functionally significant group (20). The human genome encodes 518 protein kinases, which are further categorized into serine/threonine kinases, tyrosine kinases, and tyrosine kinase-like kinases (21). Dysregulation of kinase activity is frequently implicated in the pathogenesis of diseases such as cancer and inflammatory disorders, making them prominent targets for therapeutic intervention (22). Over 250 kinase inhibitors are currently in clinical trials, and more than 30 have gained regulatory approval, underscoring the growing relevance of this enzyme family in targeted drug development (23). It has been reported that the GIKome modulate key pathological processes in sepsis, such as inflammation, immune activation, cell survival, energy metabolism, and vascular integrity (5–11). Notably, the calcium channel inhibitor CM4620 (Auxora™) has demonstrated therapeutic potential in acute pancreatitis with systemic inflammatory response syndrome (SIRS) (24, 25). with ongoing trials evaluating its efficacy and safety (26). While these targets hold significant therapeutic potential for mitigating morbidity and mortality, they remain underexplored in sepsis treatment.

On the one side, the development of druggable targets, particularly the GIKome, has been a cornerstone of drug design, driving breakthroughs across various diseases. On the other side, emerging evidence highlights the therapeutic promise of gut microbial metabolites in sepsis, such as short-chain fatty acids (SCFAs) and flavonoids, though their mechanisms and clinical applications are still in nascent stages (27). Despite the essential role of the GIKome in modulating pathological process in sepsis, their therapeutic potential remains underexplored in this context. Moreover, while gut microbial metabolites such as short-chain fatty acids and flavonoids have shown promise in regulating host immunity, the molecular mechanisms linking them to druggable host targets are poorly understood. To address this gap, the present study attempts to investigate the interactome between gut microbial metabolites and the most widely drug-targeted protein families to unveil novel therapeutic strategies for sepsis. Specifically, we applied a network-based systems biology framework, leveraging structure-based virtual screening strategy, genetics-driven approaches, and multi-omics profiling, to systematically identify therapeutic targets within the druggable GIKome and map potential gut microbial metabolite-GIK interactions in sepsis. Our findings offer new insights into gut microbial metabolite-based therapeutic strategies targeting the human GIKome for sepsis treatment.

## 2 Materials and methods

### 2.1 Data source

**Figure 1:**
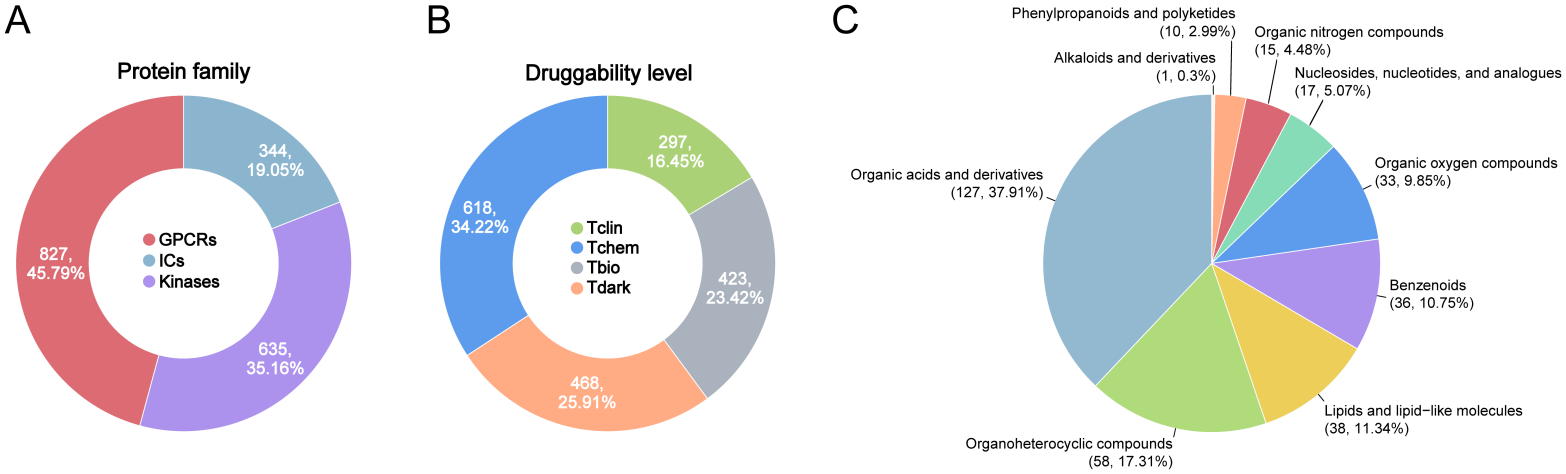
Overview of druggable GIKs and gut microbial metabolites in this study. **(A)** Classification of druggable GIKs into GPCRs, ICs, and kinases. **(B)** Categorization of druggable GIKs into four developmental stages: Tclin, Tchem, Tbio, and Tdark. **(C)** Distributions of human gut microbial metabolites by chemical classes.

The datasets used for genetics-driven analysis are detailed in **Supplementary Table S1**. Summary statistics for genome-wide association studies (GWAS) on sepsis-related outcomes were obtained from the UK Biobank (28) and the FinnGen (29), covering five sepsis-related endpoints. The druggable GIK list was derived from the Pharos database, an integrative resource developed as part of the NIH-funded IDG program, which systematically catalogs druggable targets (30). A total of 1,806 druggable GIKs were identified, including 827 GPCRs, 344 ICs, and 635 kinases (**Figure 1A**; **Supplementary Table S2**). These targets were classified into four developmental stages (**Figure 1B**). Tclin includes targets with approved drugs, while Tchem comprises proteins without approved drugs but have been shown to bind small molecules with high potency. Tbio includes targets with experimental Gene Ontology (GO) annotations or those meeting at least two of the following: a fractional publication count > 5, three or more Gene Reference Into Function (NCBI GeneRIF) annotations, or ≥ 50 commercial antibodies listed in Antibodypedia. The fourth category, Tdark, comprises proteins manually curated at the primary sequence level in UniProt but not meeting the criteria for Tclin, Tchem, or Tbio. To investigate the genetic regulation of these druggable GIKs, expression quantitative trait locus (eQTL) data were obtained from the eQTLGen Consortium (31), while protein quantitative trait locus (pQTL) data were sourced from a large-scale proteomic study based on the Icelandic cohort (32). Notably, there was no overlap between the populations analyzed for sepsis-related traits and those studied for the druggable GIKs, ensuring the validity and independence of the genetic associations.

A curated list of 335 metabolites derived from 158 human gut bacterial strains was compiled from recent *in vitro* studies, excluding those identified in colonized mouse models (33). These metabolites are specific to human gut microbiota and duplicate entries were removed based on PubChem ID and chemical name (**Figure 1C**; **Supplementary Table S3**). The metabolic profiles of these 158 bacterial strains were systematically reanalyzed, and the relative abundances of the 335 metabolites, expressed as log_2_ fold change (log_2_FC) compared to germ-free controls, were extracted from the dataset reported by Qiu et al. as presented in **Supplementary Table S4** (34). Additionally, GWAS data encompassing 1,400 plasma metabolites were retrieved from the NHGRI-EBI GWAS Catalog (35) and the 335 gut bacterial metabolites were matched using SMILES, INCHIKEY, or PubChem ID.

### 2.2 Mendelian randomization analysis

To evaluate the causal effects of druggable GIKs and gut microbial metabolites on sepsis, we conducted MR analyses using high-confidence single nucleotide polymorphisms (SNPs) as instrumental variables (IVs). IV selection was based on three stringent criteria: (1) genome-wide significance (*p* < 1×10^-8^); (2) linkage disequilibrium (LD) clumping (R^2^ < 0.1) using the 1000 Genomes Project V3 reference panel and PLINK (v1.9) (36); and (3) an F-statistic > 10 to ensure sufficient instrument strength. The F-statistic was calculated as: R^2^ = 2×MAF×(1−MAF)×β^2^, F = R^2^×(n-k-1)/k×(1-R^2^), where MAF represents the minor allele frequency, n the sample size, and k the number of IVs. For causal inference, we applied the Wald ratio estimator for single-IV analyses and the Inverse-Variance Weighted (IVW) method as the primary approach for multiple IVs (IVs ≥ 2). Additionally, we performed robustness checks using MR Pleiotropy RESidual Sum and Outlier (MR-PRESSO, IVs > 3), Bayesian weighted Mendelian randomization (BWMR, IVs ≥ 3) (37), Weighted Median (WM, IVs ≥ 3), and Maximum Likelihood (MaxLik, IVs ≥ 3). To assess horizontal pleiotropy, we conducted MR-Egger intercept tests and MR-PRESSO global tests. All MR analyses were implemented using the TwoSampleMR and MRPRESSO packages in R. This study was conducted in accordance with the STROBE-MR checklist (38).

### 2.3 Colocalization analysis

For druggable GIKs that demonstrated significant associations in the preliminary MR analyses, we conducted colocalization analysis using the coloc package in R (39). The prior probabilities were set to P_1_ = 1×10^-4^, P_2_ = 1×10^-4^, and P_12_ = 1×10^-5^, reflecting the probabilities of a SNP being associated with GIK expression, the outcome, or both, respectively. The posterior probabilities were estimated for five hypotheses: PPH_0_, indicating no association with either GIK expression or the outcome; PPH_1_, association with GIK expression but not the outcome; PPH_2_, association with the outcome but not GIK expression; PPH_3_, association with both GIK expression and outcome but with distinct causal variants; and PPH_4_, association with both GIK expression and outcome with a shared causal variant. The SNP most strongly associated with exposure (lowest *p*-value) was selected as the reference variant, and variants within a ± 100 kb window were included in the analysis. GIKs with a PPH_4_ > 0.7 were considered to provide high support for colocalization, while a PPH_4_ between 0.5 and 0.7 was interpreted as medium support.

### 2.4 Summary data-based MR analysis

To account for the intricate LD structure of the genome, in which the most strongly associated variant may not necessarily be the causal one, we employed SMR analyses (40). This approach integrates GWAS data for sepsis with eQTL data, facilitating the prioritization of putative causal variants whose effects are mediated through gene expression (41). To further dissect the underlying genetic architecture, we performed the heterogeneity in dependent instruments (HEIDI) test, which evaluates whether the observed association arises from pleiotropy or LD between distinct genetic variants. A HEIDI test *p*-value < 0.05 was considered indicative of significant LD-driven associations, warranting caution in causal inference.

### 2.5 Multi-omics analysis

To investigate the molecular targets underlying sepsis onset and progression, we assembled a set of transcriptomic (microarray, bulk RNA sequencing [RNA-seq], and single-cell RNA-seq [scRNA-seq]) and proteomic datasets derived from human blood samples. These datasets encompass comparisons between sepsis patients and healthy controls, as well as between survivors and non-survivors. In total, 37 datasets were compiled and differential expression analyses of druggable GIKs were systematically conducted across all datasets (**Supplementary Table S5**). For studies that reported differentially expressed genes (DEGs) in their original publications, we directly extracted the data from primary or **Supplementary Materials**. For datasets lacking precomputed differential expression results, raw microarray or RNA-seq data were retrieved from the Gene Expression Omnibus (GEO) and analyzed using GEO2R (42). To ensure consistency, DEGs were identified based on a standardized threshold of adjusted *p* < 0.05 and |log_2_FC| > 0.5. The strength of multi-omics evidence for each GIK was determined by the number of datasets in which it exhibited significant differential expression.

A publicly available scRNA-seq dataset (SCP548) was retrieved from the Single Cell Portal, comprising blood samples from 19 healthy individuals and 29 septic patients (43). The healthy control group included 19 individuals without clinical signs of infection. Samples were obtained from age-, sex-, and ethnicity-matched healthy donors through Research Blood Components (Massachusetts, USA) and from four previously enrolled patients at 2–3 months post-recovery. All controls had no immunodeficiency, autoimmune disease, or immunosuppressive therapy at the time of sampling. We obtained the raw count matrix for total peripheral blood mononuclear cells (PBMCs, n = 106,545) and dendritic cells (n = 19,806) across all subjects. Quality control and downstream analyses were performed using the Seurat package (44) in R, following the methodology described in the original study (43) Cell type annotation for each cluster was assigned based on the provided metadata. Differential expression analysis between septic and control groups was conducted within each cell type using the Wilcoxon rank-sum test (Seurat FindAllMarkers function) with default parameters. The AddModuleScore function from the Seurat package with default settings was used to calculate cell activity scores, including T cell, B cell, and monocyte (45).

### 2.6 Molecular docking-based virtual screening procedure

As the first step, we identified potential binding pockets for molecular docking between gut microbial metabolites and sepsis-related GIKs (termed sepGIKs, determined by genetics-driven and multi-omics evidence). Large-scale benchmarks have demonstrated that cavity-focused docking improves both hit rates and the accuracy of blind docking. Therefore, we employed the CB-Dock online server (46) developed by Liu et al., which enhances docking precision by predicting protein binding sites using a curvature-based cavity detection approach (CurPocket) (47). This method enables the identification of binding sites, determination of their geometric center and dimensions, and customization of the docking box for each GIK. In total, 570 druggable pockets were identified across 114 AlphaFold2-predicted (48) sepGIK structures (**Supplementary Table S6**). Then, molecular structures were prepared using AutoDock utilities, followed by molecular docking with AutoDock Vina (49). To enhance the search space coverage, the exhaustiveness parameter was set to 30. For each docking simulation, the top-ranked (1^st^) binding conformation and corresponding docking score were retained for further comparison (**Supplementary Table S13**).

### 2.7 Metabolite-sepGIK interaction network

To systematically characterize interactions between gut microbial metabolites and sepGIKs, we constructed a metabolite-sepGIK interaction network based on molecular docking-derived binding affinities. The network comprises four distinct layers (1): Nodes (n = 449), including 335 metabolites and 114 sepGIKs, along with their respective classifications; (2) Edges (n = 449), representing metabolite-sepGIK interactions, including the 1^st^ sepGIK for each metabolite and the 1^st^ metabolite for each sepGIK; (3) Genetics-driven and multi-omics evidence, supporting the involvement of sepGIKs in sepsis; (4) Microbial associations, capturing the number of bacterial strains linked to each metabolite. The network was visualized using Cytoscape 3.9.1 to facilitate interpretation of metabolite-sepGIK relationships.

### 2.8 Molecular dynamics simulation

The molecular docking-derived complex was prepared following the guidelines outlined in the GROMACS tutorial. System setup was conducted via CHARMM-GUI, incorporating TIP3P water and 150 mM NaCl to mimic physiological conditions. MD simulation was performed using GROMACS 2022.2, employing a 2 fs timestep over 100 ns, with standard force field parameters. Equilibration was carried out under NVT and NPT ensembles. To assess binding stability and energetic contributions, MM/PBSA free energy calculations were conducted using gmx_MMPBSA (50), based on stable 10 ns during the simulation. Structural stability and interaction strength of the protein-ligand complex were evaluated through MD trajectory analysis, considering root-mean-square deviation (RMSD), root-mean-square fluctuation (RMSF), solvent-accessible surface area (SASA), radius of gyration (Rg), and hydrogen bond (HB) formation. These parameters were computed using standard GROMACS commands (gmx rms, gmx rmsf, gmx hbond, gmx SASA, and gmx gyrate), with data visualization facilitated by DuIvyTools 0.6.0 (https://duivytools.readthedocs.io/en/latest/index.html). Finally, the gmx trjconv module was employed to extract protein-ligand complex structures at specific time points for further analysis.

### 2.9 Microscale thermophoresis assay

Indole-3-lactic acid (ILA; MCE, USA) at varying concentrations was incubated with fluorescently labeled 6-phosphofructo-2-kinase/fructose-2,6-bisphosphatase 2 (PFKFB2) (Abcam, Cambridge, UK) for 30 minutes at room temperature. The binding interaction between ILA and PFKFB2 was quantified using a Monolith NT.115 instrument (NanoTemper Technologies, München, Germany). The dissociation constant (Kd) was determined using NT Analysis software (51).

### 2.10 Functional enrichment analysis

GO and KEGG enrichment analyses of 114 sepGIKs were conducted with the clusterProfiler R package. The results were visualized by the ggplot2 R package. The enrichment results are shown in **Supplementary Figure S4**.

### 2.11 Animals and treatment

Eight-week-old male C57BL/6J mice (22 ± 2 g) were obtained from Vital River Laboratory Animal Technology (Beijing, China). All animals were housed in a specific pathogen-free (SPF) environment under controlled conditions (12-hour light/dark cycle, 20-25°C, 50 ± 5% humidity) at the Experimental Animal Center of Chongqing Medical University (Chongqing, China), with ad libitum access to standard chow and water. Following a one-week acclimatization period, mice were randomly assigned to three groups (n = 6 per group): Sham, cecal ligation and puncture (CLP), and CLP + ILA. Mice received daily gavage of either corn oil (0.1 mL/20 g) or ILA (20 mg/kg) for seven consecutive days (52). The CLP model was established as previously described (53). Under anesthesia, a midline laparotomy (~1 cm) was performed to expose the cecum, which was ligated with 3–0 silk sutures and punctured twice with a 20-gauge needle near the distal end. Gentle pressure was applied to extrude fecal matter into the peritoneal cavity before repositioning the cecum and closing the incision. Mice in the Sham group underwent identical surgical procedures without ligation or puncture. Postoperatively, all mice received subcutaneous resuscitation with 1 mL of pre-warmed (37°C) sterile saline. For survival experiments (n = 12 per group), an aggravated CLP model was constructed (54). Sepsis severity was assessed using the Murine Sepsis Score (MSS), as established by Shrum et al. (55). At 24 hour post-CLP, mice were euthanized via sodium pentobarbital overdose, and blood and organ samples were collected. Mouse blood samples were collected via retro-orbital bleeding into sterile, serum collection tubes without anticoagulants (Thermo Fisher Scientific, USA). The samples were allowed to clot at room temperature for 2 hours, followed by centrifugation at 3,000 rpm for 10 minutes at 4°C. The supernatant serum was carefully collected and stored at -80°C until further analysis. All animal procedures complied with national ethical guidelines and were approved by the Ethics Committee of the Second Affiliated Hospital of Chongqing Medical University.

### 2.12 Hematoxylin and eosin staining

Heart, liver, lung, and kidney tissues were fixed in 10% paraformaldehyde for at least 48 hours, embedded in paraffin, and sectioned at a thickness of 4 µm. The sections were deparaffinized in xylene, rehydrated through a graded ethanol series, and subjected to H&E staining. After staining, sections were dehydrated, cleared, and mounted. Histopathological changes were assessed using an optical microscope (Olympus, Japan).

### 2.13 Inflammatory cytokine and lactic acid detection

Serum levels of TNF-α, IL-1β, and IL-6 were measured using enzyme-linked immunosorbent assay (ELISA) kits from Thermo Fisher Scientific (Cat# BMS607–3 for TNF-α, BMS6002–2 for IL-1β, and BMS603–2 for IL-6), according to the manufacturer’s protocols. The assay ranges were 31.3-2,000 pg/mL for TNF-α and IL-6, and 7.8–500 pg/mL for IL-1β. Kits for lactate production assay (Cat# A019-2-1; assay range: 0–6 mmol/L) were purchased from Nanjing Jiancheng (Nanjing, China).

### 2.14 Statistical analysis

Data are presented as the mean ± standard deviation (SD). Statistical analyses were conducted using SPSS (version 26.0) and R (version 4.3.1). Comparisons between two groups were performed using *t*-test, while differences among multiple groups were analyzed by one-way analysis of variance (ANOVA), followed by *post hoc* tests where appropriate. Survival curves were generated using the Kaplan-Meier (KM) method and compared with the log-rank test. A *p*-value < 0.05 was considered statistically significant.

## 3 Results

### 3.1 A systems biology framework prioritizes associations between gut microbial metabolites and the druggable GIKome in sepsis

This study presents a multi-layered systems biology framework to prioritize gut microbial metabolites associated with the druggable GIKome in sepsis. Our approach integrates genetic, multi-omics, and computational methodologies with experimental validation and follows a four-stage workflow. Stage 1 aimed to identify a high-confidence set of sepsis-associated and druggable GIKs (sepGIKs) by integrating genetic causality and multi-omics support. We first applied Mendelian randomization-based approaches, including MR_eQTL_, MR_pQTL_, SMR, and colocalization, to infer causal links between genetic variants regulating GIK expression and sepsis risk. This allowed us to prioritize GIKome genes with genetic evidence of involvement in sepsis. Additionally, we employed differential expression profiling across bulk RNA-seq, microarray, scRNA-seq, and proteomic datasets to identify potential GIKs in sepsis. These genetically linked and differentially expressed GIKs were designated as sepGIKs and subjected to downstream analysis. Stage 2 aimed to identify gut microbial metabolites with potential causal roles in sepsis using MR analysis, based on a comprehensive metabolite library derived from human gut strains (33). This step provided a prioritized list of bioactive compounds with potential systemic impact on host pathophysiology during sepsis. Stage 3 served as a bridge linking the host and microbial layers by predicting metabolite-sepGIK interactions through structure-based virtual screening. This enabled the construction of a functional interaction network between candidate metabolites and sepGIKs. Stage 4 validated the biological relevance of key predicted metabolite-target interactions using a cecal ligation and puncture (CLP) mouse model of sepsis, thus providing experimental support for therapeutic insights derived from the preceding in silico analyses. This stepwise approach establishes a translational pipeline from therapeutic target discovery to microbial metabolite-host interaction mapping and *in vivo* validation, offering a novel strategy for therapeutic development in sepsis. A comprehensive schematic of the workflow is provided in **Supplementary Figure S1**.

### 3.2 Genetics-driven and multi-omics approaches reveal candidate GIK targets in sepsis

**Figure 2:**
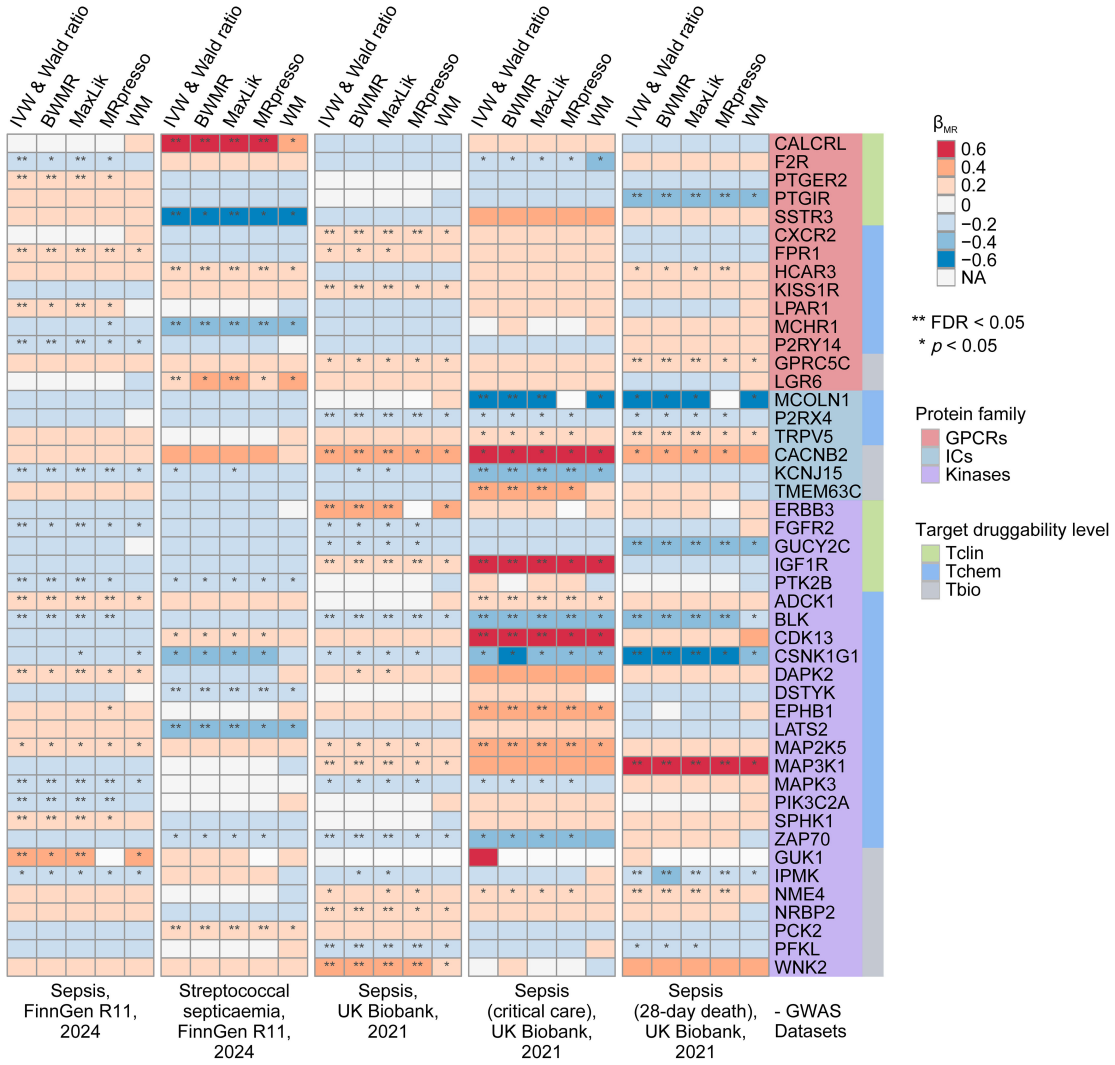
Prioritization of druggable GIKs through MR analysis. In total, five sepsis GWAS datasets from the UK Biobank and FinnGen were used. The eQTL data for druggable GIKs were obtained from the eQTLGen Consortium. β > 0 indicates that increased expression of a GIK is associated with a higher likelihood of sepsis.

In our preliminary MR analyses, we investigated the relationship between 1,806 druggable GIKs and sepsis. From an eQTL dataset derived from human blood, we identified 781 GIKs with IVs meeting stringent criteria (*p* < 5×10^-8^, F-statistic > 10). These included 163 GPCRs, 159 ICs, and 459 kinases (**Supplementary Table S7**). We further identified 46 GIKs that were significantly associated with at least one of five distinct sepsis outcomes (FDR_IVW_ < 0.05; **Figure 2**; **Supplementary Table S8**). Among these, five GIKs, including BLK (B lymphoid tyrosine kinase), ADCK1 (AarF domain-containing kinase 1), KCNJ15 (potassium inwardly-rectifying channel subfamily J member 15), MAP3K1 (mitogen-activated protein kinase kinase kinase 1), and IGF1R (insulin-like growth factor 1 receptor), were consistently validated across at least two sepsis outcomes (FDR_IVW_ < 0.05; **Figure 2**). Notably, upregulation of ADCK1, IGF1R, and MAP3K1 at the transcriptional level was found to predict higher sepsis risk, while increased expression of BLK and KCNJ15 correlated with lower risk (**Figure 2**). For example, elevated expression of the kinase BLK was strongly associated with a reduction in 28-day mortality risk in sepsis (β_IVW_ = -0.295, FDR = 2.98×10^-12^; **Supplementary Table S8**).

To investigate the underlying genetic mechanisms, we examined the potential role of shared genetic variants driving these associations. We identified significant colocalization evidence for MCOLN1 (mucolipin 1) and IGF1R in relation to sepsis in critical care, with posterior probability of colocalization (PPH_4_) values of 0.705 and 0.732, respectively (**Supplementary Figure S2**; **Supplementary Table S9A**). Additionally, moderate colocalization evidence was observed for MAP2K5 (mitogen-activated protein kinase kinase 5), PIK3C2A (phosphatidylinositol-4-phosphate 3-kinase catalytic subunit type 2 alpha), and DSTYK (dual serine/threonine and tyrosine protein kinase) with sepsis outcomes (PPH_4_ > 0.5; **Supplementary Table S9A**).

Subsequently, we performed SMR analyses, which provided robust causal evidence for 14 of the 46 identified GIKs associated with sepsis (*p* < 0.01; **Supplementary Figure S3**; **Supplementary Table S9B**). Importantly, the HEIDI test confirmed that all observed associations were independent of linkage disequilibrium effects (*p* > 0.05; **Supplementary Table S9B**).

Finally, pQTL replication analyses at the protein level corroborated our findings (**Supplementary Table S9C**), revealing that higher circulating levels of ERBB3 (Erb-B2 receptor tyrosine kinase 3) were associated with increased sepsis risk (β_IVW_ = 0.314, *p* = 1.09 × 10^-2^), while elevated NME4 (NME/NM23 nucleoside diphosphate kinase 4) levels were linked to higher 28-day mortality (β_IVW_ = 0.448, *p* = 6.78 × 10^-3^).

**Figure 3:**
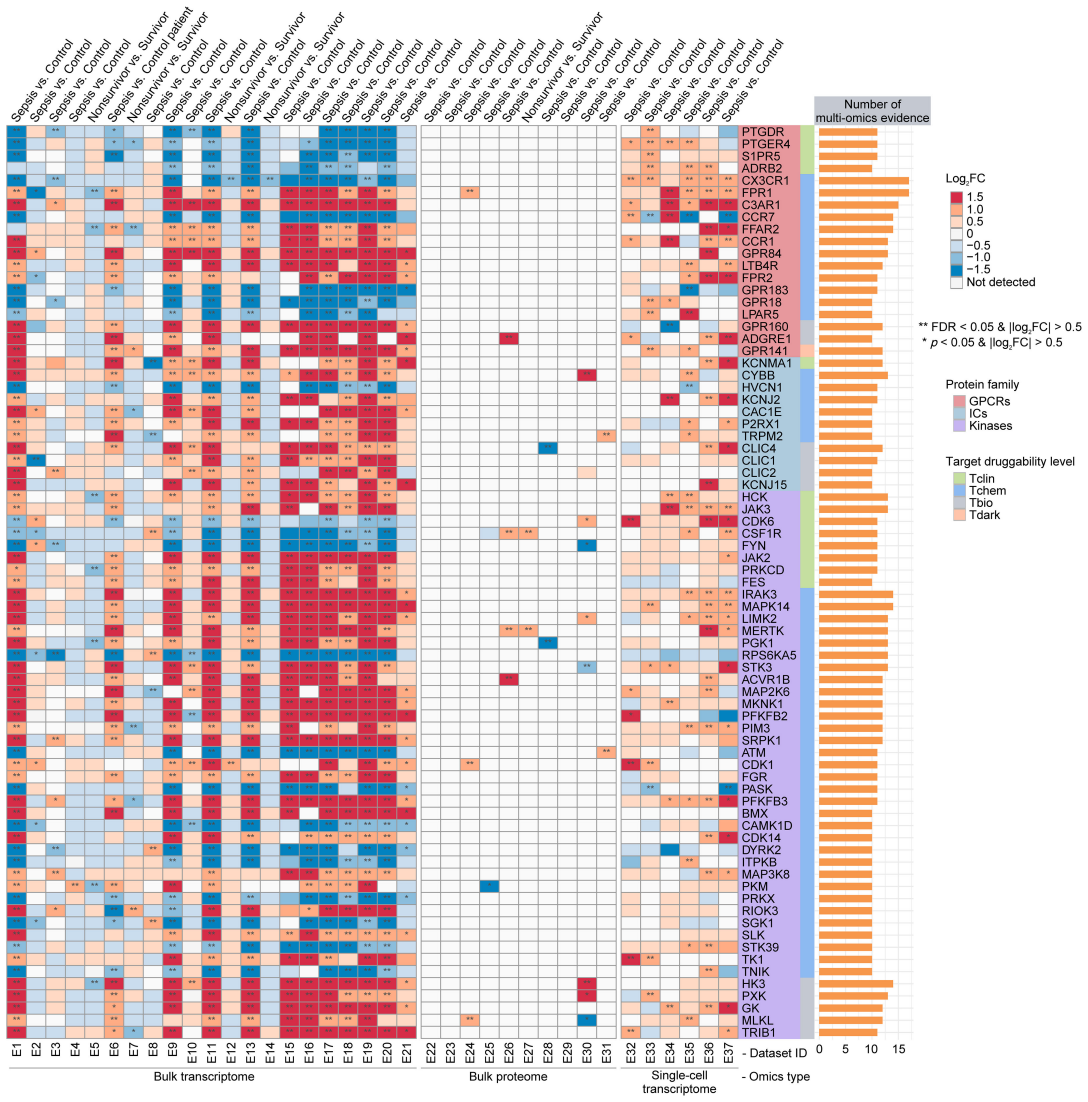
Prioritization of druggable GIKs through multi-omics analysis. A comprehensive analysis of 37 sepsis transcriptomic and proteomic datasets identified druggable GIKs that are differentially expressed between sepsis and control states, as well as between survivors and non-survivors. The number of datasets providing multi-omics evidence for each GIK is represented by the stacked bars.

To identify potential sepGIKs, we integrated bulk and single-cell transcriptomic as well as proteomic data from blood samples of septic patients. Leveraging 37 manually curated sepsis transcriptomic and proteomic datasets, we performed differential expression analyses on 1,806 druggable GIKs, comparing disease states (sepsis vs. control) and clinical outcomes (survivor vs. non-survivor) (**Supplementary Tables S5**, **S10**). This analysis revealed 75 GIKs differentially expressed in at least 10 datasets, comprising 19 GPCRs, 11 ICs, and 45 kinases (**Figure 3**; **Supplementary Table S11**). Among these, three GPCRs, including CX3CR1 (C-X3-C motif chemokine receptor 1), FPR1 (formyl peptide receptor 1), and C3AR1 (complement component 3a receptor 1), emerged as the most promising therapeutic candidates, exhibiting differential expression in at least 15 independent datasets (**Figure 3**).

Collectively, these genetics-informed and multi-omics-driven insights underscore the functional relevance of the druggable GIKome in sepsis and highlight novel therapeutic avenues for intervention.

### 3.3 Discovery of human gut microbial metabolite-sepGIK interactome via a molecular docking-based virtual screening pipeline

We sought to identify interactions between gut microbial metabolites and sepGIKs via a virtual screening approach based on cavity-focused docking. This involved screening 335 metabolites derived from 158 human gut bacteria strains (33) (**Supplementary Table S3**) against 114 sepGIKs (**Figures 2**, **3**). In total, 190,950 metabolite-sepGIK pairs were evaluated (**Supplementary Table S12**), encompassing 114 sepGIKs (570 ligand-binding pockets; **Supplementary Table S6**) and 335 metabolites. Our analysis revealed that sepGIKs were primarily regulated by nucleosides, nucleotides, and their analogues, as well as phenylpropanoids and polyketides, exhibiting mean binding affinities of -6.82 kcal/mol and -6.25 kcal/mol, respectively (**Supplementary Figure S5**).

**Figure 4:**
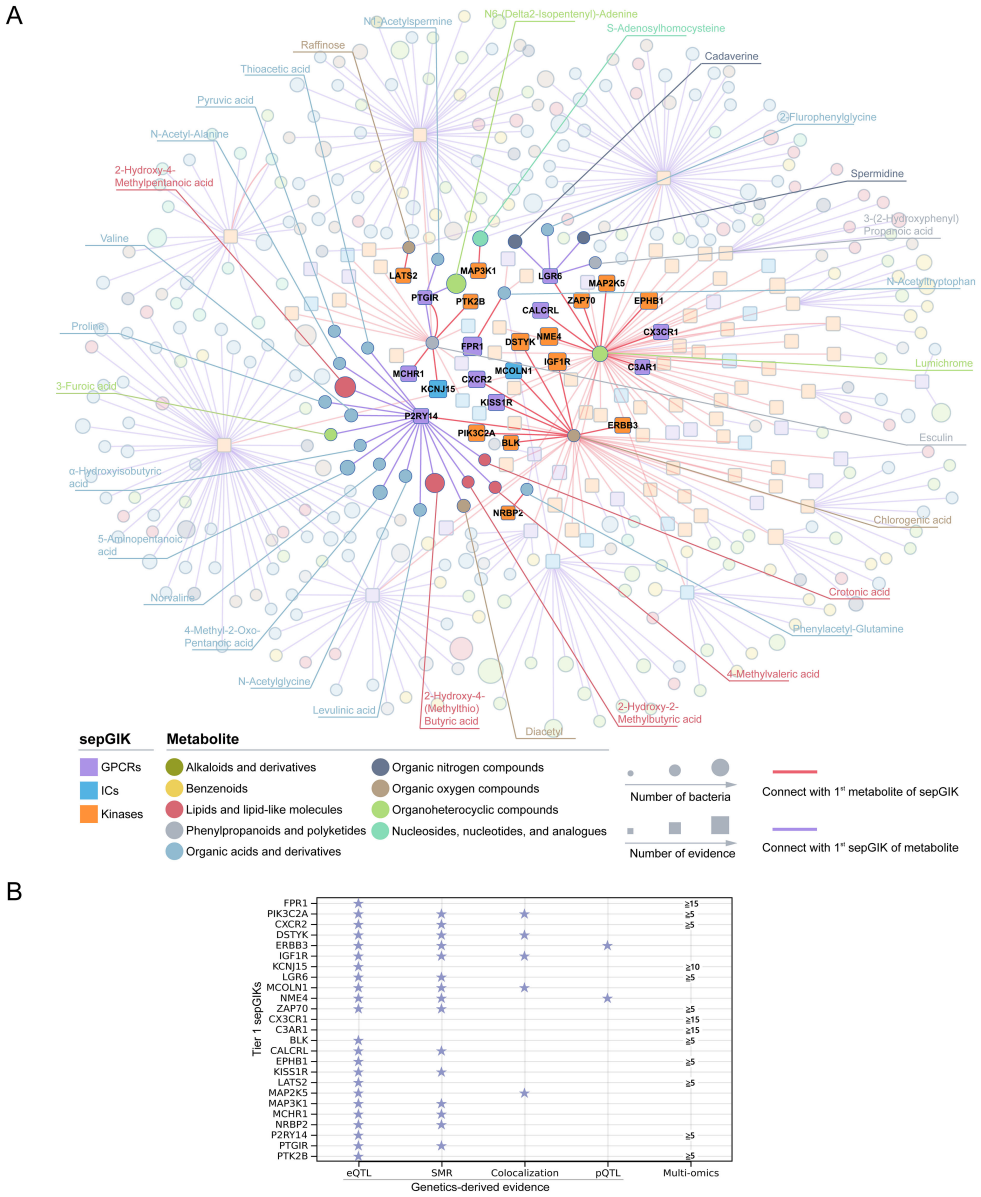
Molecular docking-based discovery of human gut microbial metabolite-sepGIK interactome. **(A)** An integrated network illustrates the gut microbial metabolite-sepGIK interactome. A docking score (edge) connecting the 1^st^ metabolite (shown in red) of 114 sepGIKs or the 1^st^ sepGIK (shown in purple) of 335 metabolites. Metabolite and sepGIK are depicted as circle and rectangle nodes, respectively. Protein family class of sepGIKs and chemical class of metabolites are indicated with different colors. The size of the sepGIK node is proportional to the number of multi-omics or genetic evidence, while the size of the metabolite node is proportional to the number of bacteria strains with higher metabolite abundance (|log_2_FC| ≥ 2). Tier 1 sepGIKs and their paired metabolites are highlighted. **(B)** Summary of Genetics-driven and multi-omics evidence for Tier 1 sepGIKs.

Subsequently, we investigated the 1^st^ sepGIK for each metabolite and the 1^st^ metabolite for each sepGIK, generating a network of 449 predicted metabolite-sepGIK pairs, linking 114 sepGIKs with 335 metabolites (**Figure 4A**; **Supplementary Table S14**). To further dissect these interactions, we classified sepGIKs into two tiers based on Genetics-driven and multi-omics evidence. A Tier 1 target is defined as one supported by at least two genetically derived pieces of evidence (MR_eQTL_, MR_pQTL_, SMR, and colocalization) or by one such piece of evidence coupled with differential expression in at least five sepsis transcriptomic or proteomic datasets, or by differential expression across 15 or more datasets (**Figure 4B**; **Supplementary Table S14**). Conversely, Tier 2 targets are supported by at least one genetic evidence or differential expression in at least 10 datasets (**Supplementary Table S14**). Further analysis indicated that Tier 1 sepGIKs included 50 metabolite-sepGIK pairs, linking 25 sepGIKs to 31 human gut microbial metabolites (**Figure 4A**; **Supplementary Table S14**). Tier 2 sepGIKs included 89 sepGIKs (399 pairs; **Supplementary Table S14**). Collectively, these findings provide a comprehensive network linking human gut microbial metabolites with sepGIKs.

### 3.4 MR analysis uncovers sepsis-relevant gut microbial metabolites

**Figure 5:**
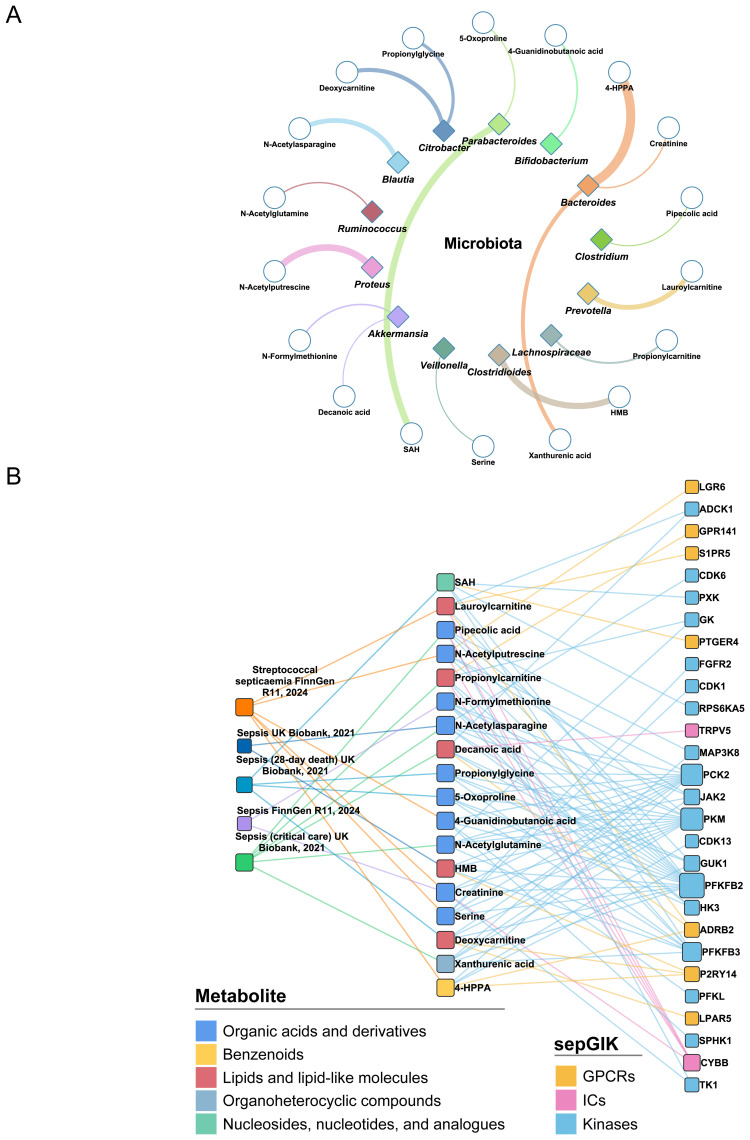
Identification of sepsis-relevant gut microbial metabolites using MR analysis. **(A)** Gut microbial origins of 18 MR-prioritized metabolites. Only the bacteria genera that have the most abundance of metabolites are shown. The color of shape and lines are based on bacteria genus types. The line width indicates the abundance of metabolite in the bacteria. HMB, 2-Hydroxy-4-(methylthio)butanoic acid; SAH, S-Adenosylhomocysteine; 4-HPPA, 4-Hydroxyphenylpyruvate. **(B)** Associations between MR-prioritized metabolites and sepGIKs. In total, 18 gut microbial metabolites supported by MR analysis (*p*_IVW < 0.05) on five sepsis GWAS datasets interact with 28 sepGIKs.

To elucidate causal gut microbial metabolites linked to sepsis, we performed MR analysis on 335 metabolites, identifying 94 metabolites with valid IVs (*p* < 5×10^-8^, F-statistic > 10) from GWAS of plasma metabolites (**Supplementary Table S15A**). Of these, 18 gut microbial metabolites, with high abundance in gut microbiota genera (33) (**Figure 5A**; **Supplementary Table S4**), were found to be significantly associated with sepsis (*p*_IVW < 0.05; **Supplementary Figure S6**; **Supplementary Table S15B**). For instance, the asparagine derivative N-acetylasparagine exhibited significantly elevated levels in *Blautia hansenii* (log_2_FC = 6.51, relative to the germ-free control; **Supplementary Table S4**) (33). Furthermore, five metabolites, including 5-oxoproline, N-acetylasparagine, N-acetylglutamine, N-formylmethionine, and serine, were consistently linked to an increased or decreased risk of sepsis across all MR methods (*p* < 0.05; **Supplementary Figure S6**). Among these, 5-oxoproline, produced by *Parabacteroides johnsonii*, exhibited the strongest association with an increased risk of sepsis-related mortality (β_IVW_ = 0.249, *p* = 1.04×10^-5^). These findings support the existence of likely causal relationships between gut microbial metabolites and sepsis pathogenesis.

To explore potential interactions between sepsis-related gut microbial metabolites and sepGIKs, we prioritized the top five sepGIKs based on binding scores for the 18 metabolites (**Figure 5B**). This analysis revealed 28 sepGIKs, consisting of 7 GPCRs, 2 ICs, and 19 kinases. Notably, PFKFB2, differentially expressed across 12 sepsis datasets (**Figure 3**), exhibited strong binding affinity with 15 of the gut microbial metabolites (**Figure 5B**). The strongest binding interaction was observed between PFKFB2 and SAH (affinity = -9.0 kcal/mol; **Supplementary Table S13**). Furthermore, we identified N-acetylputrescine with high abundance in *Proteus* (log_2_FC = 9.62; **Supplementary Table S4**), as a candidate binding metabolite for the Tier 1 sepGIK LGR6 (leucine-rich repeat domain-containing GPCR 6) (**Figure 4B**).

### 3.5 scRNA-seq analysis implies immunosuppression-related metabolite-sepGIK pairs

**Figure 6:**
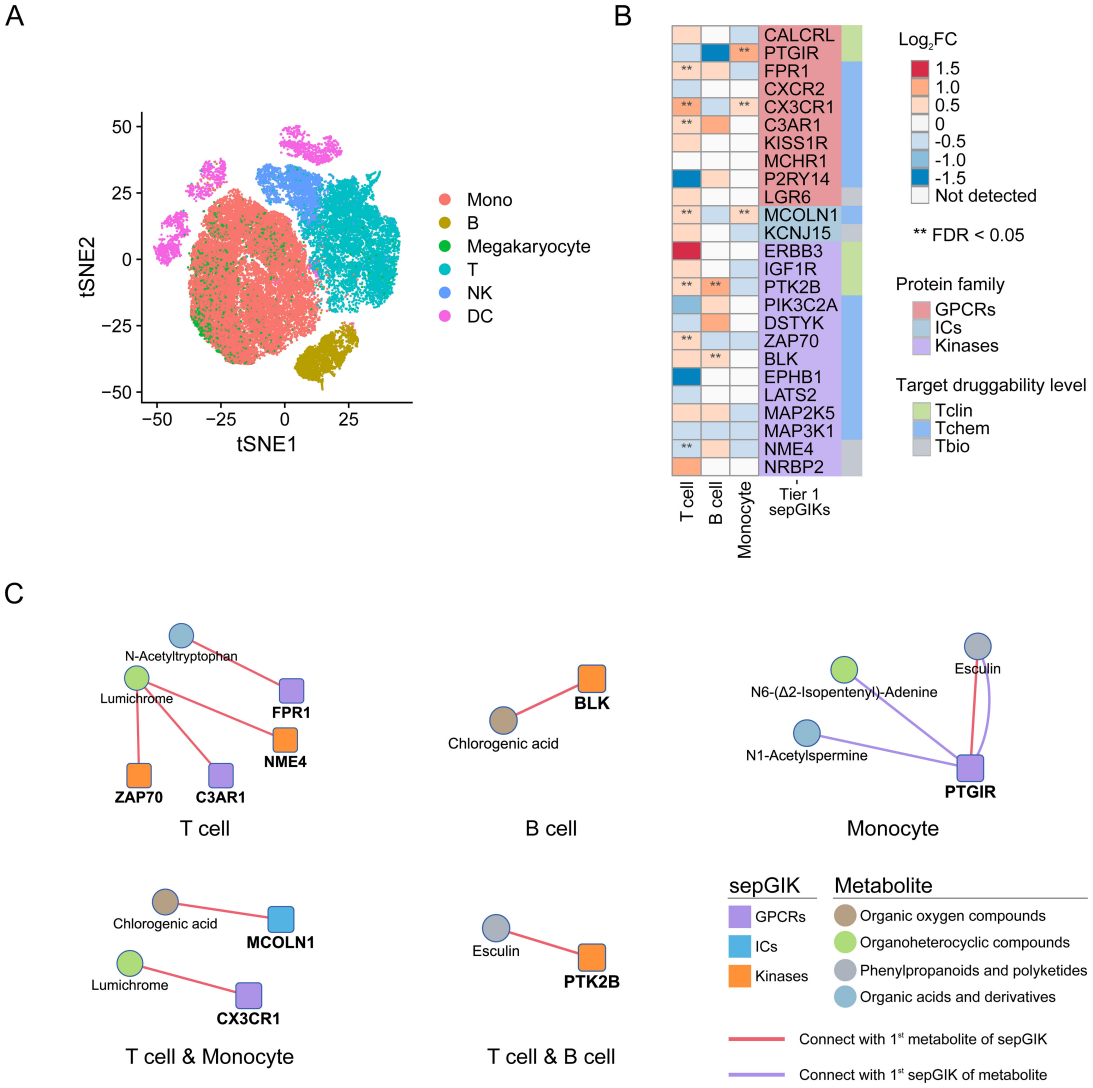
scRNA-seq-based discovery of immunosuppression-related gut microbial metabolite-sepGIK pairs. **(A)** t-distributed stochastic neighbor embedding (tSNE) plot for each cell type based on SCP548 scRNA-seq dataset. **(B)** Heatmap depicting the differential expression of Tier 1 sepGIKs across high- and low-activity immune cells, including T cell, B cell, and monocyte. **(C)** Network diagram illustrating the immunosuppression-related metabolite-sepGIK pairs based on T cell, B cell, and monocyte.

Lymphocyte activity and monocyte human leukocyte antigen-DR (mHLA-DR) expression, is often used as a measure of immune status in sepsis (45, 56). In this study, we identified several Tier 1 sepGIKs that exhibit differential expression between high- and low-activity immune cells in sepsis (FDR < 0.05; **Figure 6B**; **Supplementary Table S16**), including ZAP70 (zeta-chain-associated protein kinase 70), CX3CR1, FPR1, MCOLN1, NME4, C3AR1, PTGIR (prostaglandin I2 receptor), PTK2B (Protein Tyrosine Kinase 2 beta), and BLK. For example, CX3CR1 and MCOLN1 were downregulated in both low-activity T cells and low-HLA-DR monocytes (**Figure 6B**). As shown in **Figure 6C**, MCOLN1 was shown to interact with chlorogenic acid, while CX3CR1 exhibited the strongest binding affinity for lumichrome. In summary, these findings provide a comprehensive overview of potential gut microbial metabolite-sepGIK interactions that contribute to immunosuppression during sepsis.

### 3.6 Identification of therapeutic gut metabolites from sepsis-protective microbiota

**Figure 7:**
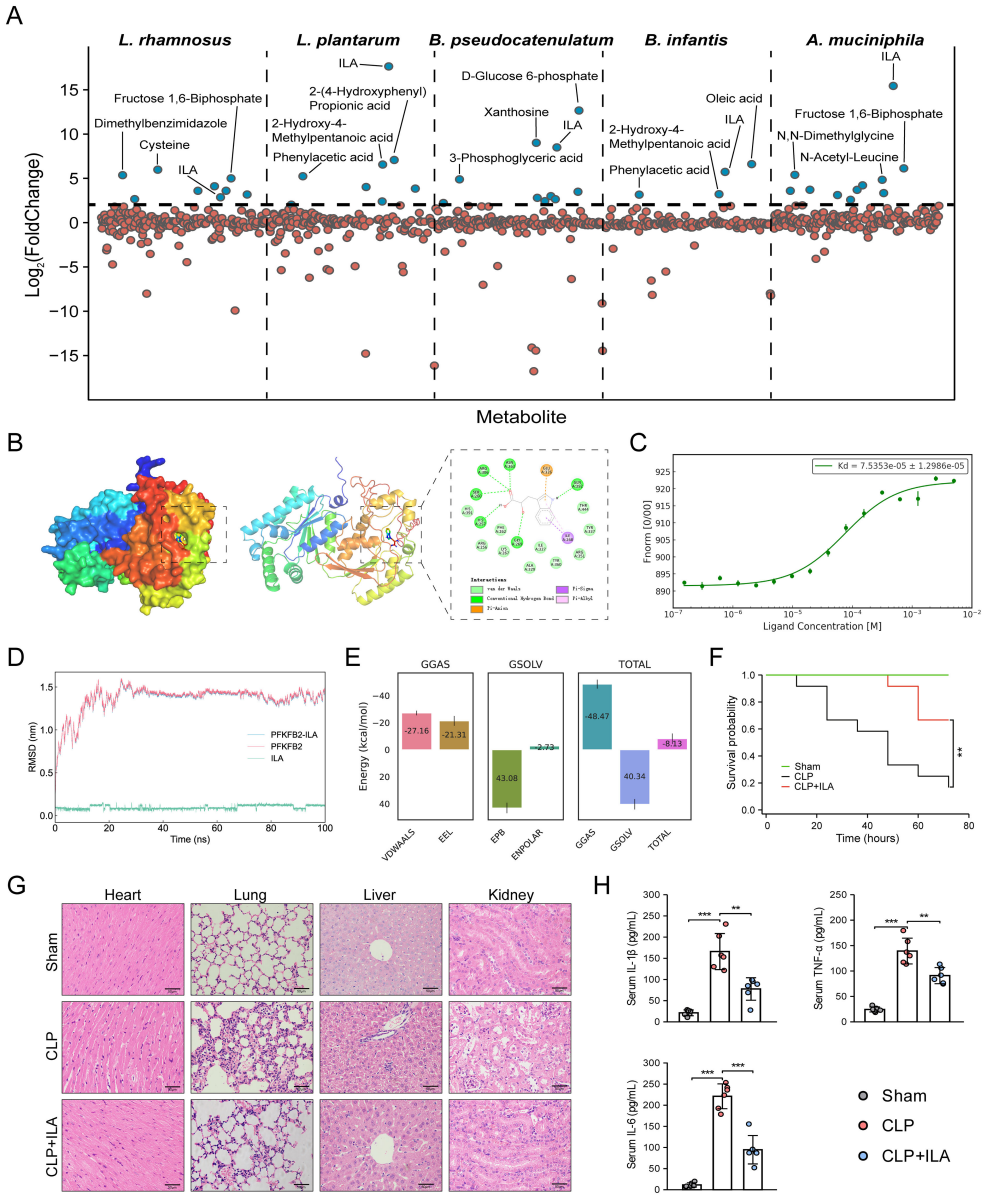
Prioritization of human gut microbial metabolites from sepsis-protective microbiota. **(A)** Abundance of gut metabolites from *L. rhamnosus*, *L. plantarum*, *B*. *pseudocatenulatum*, *B. infantis*, and *A. muciniphila*. **(B)** Visualization of ILA interaction with PFKFB2. **(C)** MST analysis for ILA binding to PFKFB2 (n = 3). **(D)** RMSD analysis over time, reflecting structural stability. **(E)** MM/PBSA energy decomposition. VDWAALS, Van der Waals interactions; EEL, electrostatic energy; EPB, polar solvation energy; ENPOLAR, nonpolar solvation energy; GGAS, gas-phase energy; GSOLV, solvation free energy; TOTAL, total binding free energy. **(F)** The KM survival curves assessed for up to 72 h. Each line represents the survival of mice in a group; 12 mice were in each group. **(G)** Representative images of HE staining exhibited the pathological alterations in multiple organs of mice, including lung, liver, kidney, and heart (Scale bar = 50 μm). **(H)** ELISA for detecting the serum levels of TNF-α, IL-1β, and IL-6. *p < 0.05, **p < 0.01, ***p < 0.001.

We next prioritized human gut metabolites derived from several specific sepsis-protective microbiota. *Lactobacillus* (*L.*) spp. and *Bifidobacterium* (*B.*) spp. are the most extensively studied genera known for their protective roles against sepsis (27). By inspecting metabolite profiles and their abundances (**Supplementary Table S4**), we identified significant elevations in 9, 8, 10, and 4 human gut metabolites in *L. rhamnosus*, *L. plantarum*, *B. pseudocatenulatum*, and *B. infantis*, respectively, each exhibiting a minimum fourfold increase (log_2_FC ≥ 2; **Figure 7A**) (33). Among these, D-glucose 6-phosphate displayed a remarkable accumulation in *B. pseudocatenulatum* (log_2_FC = 12.66), with its 1^st^ sepGIK identified as pyruvate kinase (PKM; **Supplementary Figure S7**). Beyond these classical probiotic genera, *Akkermansia muciniphila* (*A. muciniphila*) has emerged as a next-generation probiotic with compelling therapeutic potential for sepsis (57, 58). Metabolomic analysis revealed that *A. muciniphila* significantly elevated 11 human gut metabolites (log_2_FC ≥ 2; **Figure 7A**), with ILA exhibiting the highest accumulation (log_2_FC = 15.44; **Supplementary Table S4**). Strikingly, ILA was consistently enriched across all five sepsis-protective microbial species (log_2_FC ≥ 2; **Figure 7A**), suggesting a protective role in modulating host responses to sepsis. As shown in **Supplementary Figure S7**, PFKFB2 (59) is the 1^st^ sepGIK for ILA. As shown in **Figure 7B**, molecular docking demonstrated that ILA forms multiple hydrogen bonds with PFKFB2 residues including Asn263, Arg306, Ser260, His257, Gln392, and Gly269, contributing to binding stability and specificity. A π-anion interaction with Glu326 and π-alkyl/π-σ interactions with Ile268 further enhance ILA’s affinity by stabilizing the aromatic ring within the binding pocket.

To further investigate the interaction between ILA and PFKFB2, we performed MD simulation. The RMSD of the ILA-PFKFB2 complex stabilized around 30 ns, indicating a stable binding interaction (**Figure 7D**). RMSF analysis revealed that ILA had minimal impact on the flexibility of PFKFB2, likely due to its binding within an internal region with inherently low conformational mobility (**Supplementary Figure S8A**). Additional parameters, including SASA, Rg, and HB formation, further supported the stability of the complex (**Supplementary Figures S8B–D**). The Gibbs free energy landscape identified the lowest-energy conformation (**Supplementary Figure S9**), while MM/PBSA analysis confirmed the thermodynamic stability of ILA within the active site, yielding a total binding free energy of -8.13 ± 3.87 kcal/mol (**Figure 7E**). To validate these findings, we employed MST to characterize the binding affinity of ILA to PFKFB2 (**Figure 7C**). Increasing ILA concentrations led to a progressive decrease in the thermophoretic mobility of PFKFB2, producing a sigmoidal binding curve with distinct upper and lower plateaus, indicative of a specific interaction. The calculated Kd was 75.4 μM, suggesting a moderate yet specific binding affinity. Collectively, these findings establish PFKFB2 as a potential molecular target through which ILA exerts its pharmacological effects.

### 3.7 ILA protects against sepsis by attenuating inflammation and multi-organ injury

In this study, mice subjected to CLP were pretreated with three different doses of ILA (10, 20, and 40 mg/kg). First, sepsis severity was assessed according to the MSS score obtained by checking variables, including appearance, level of consciousness, activity, response to stimulus, eyes, respiration rate, and respiration quality, as established by Shrum et al. (55). The results demonstrated that ILA significantly reduced MSS scores of CLP-induced mice (**Supplementary Figure S10**). Notably, the 20 mg/kg dose of ILA conferred the most pronounced protective effect and was therefore selected for subsequent experiments. Then, we performed survival analysis. As anticipated, CLP induced a high mortality rate in mice (~80%). Notably, ILA treatment significantly improved survival outcomes (**Figure 7F**). Histopathological analysis further revealed that ILA mitigated sepsis-induced damage across multiple organs, including the heart, lungs, liver, and kidneys (**Figure 7G**). We next examined key inflammatory mediators. ILA pretreatment markedly reduced serum levels of TNF-α, IL-6, and IL-1β (**Figure 7H**), indicating a robust anti-inflammatory effect. Collectively, our findings identify ILA as a potential therapeutic agent in sepsis, conferring protection by dampening systemic inflammation and preserving organ integrity. Moreover, as the 1^st^ sepGIK of ILA, PFKFB2 plays an important immunoregulatory role in sepsis by participating in the glycolytic pathway (59). As a major end product of glycolysis, lactate serves as a practical indicator of glycolytic activity. Interestingly, our data showed that ILA pretreatment significantly reduced blood lactate levels in septic mice (**Supplementary Figure S11**), suggesting a potential inhibitory effect of ILA on glycolysis.

Considering the clinical relevance of a therapeutic setting, we further evaluated the efficacy of ILA when administered after the onset of sepsis. To this end, mice were treated with ILA at a dose of 20 mg/kg following CLP. The administration was repeated every 12 hours for three consecutive days. The results showed that post-treatment with ILA significantly alleviated sepsis severity and improved survival in CLP-induced mice (**Supplementary Figure S12**), further highlighting the therapeutic potential of ILA.

## 4 Discussion

Sepsis remains one of the most formidable challenges in modern medicine, with high mortality rates and limited therapeutic options. Traditional strategies, such as broad-spectrum antimicrobials and supportive care, have proven inadequate in the face of sepsis-induced immunological and metabolic dysregulation. Recent advances underscore the promise of biomarker-driven, personalized therapies in sepsis (60, 61). However, despite progress in targeted approaches, such as cytokine inhibitors, monoclonal antibodies, and recombinant immunomodulatory proteins, their clinical translation remains hindered by the heterogeneous nature of sepsis pathophysiology and interpatient variability in treatment responses (3, 61). The identification of metabolites produced by probiotic bacteria, termed metabiotics, has gained significant traction due to their profound anti-inflammatory, anti-obesogenic, and immunomodulatory effects. Postbiotics, in conjunction with dietary factors, exert regulatory influence over both physical and mental health, as the microbiota interact with host cells either directly or via the secretion of bioactive metabolites (62). Recent studies highlight the gut microbiota’s pivotal role in modulating systemic inflammation and organ dysfunction during sepsis. Microbiota-targeted interventions, including fecal microbiota transplantation (FMT), selective digestive decontamination (SDD), and microbiota-derived metabolites, have shown potential in restoring intestinal barrier integrity and mitigating systemic inflammation (27, 63). However, they remain underexplored in sepsis treatment. To overcome this, the integration of advanced tools, such as multi-omics profiling, high-resolution metabolomics, and genetics-driven biomarker discovery, holds promise in identifying actionable therapeutic targets (64). A multidisciplinary approach that converges microbiome science, precision medicine, and systems biology is imperative to unravel the systemic complexities of this life-threatening condition.

One of the key contributions of this study is the successful identification of 114 potential sepGIKs. Based on genetically derived and multi-omics-derived evidence, we categorized these targets into Tier 1 and Tier 2 groups. Notably, a substantial proportion of these targets have been previously reported in independent studies, reinforcing the validity of our predictions and highlighting the potential of these targets for further mechanistic and translational investigations. For example, three GPCRs, CX3CR1, FPR1, and C3AR1, exhibiting differential expression in at least 15 independent datasets (**Figure 3**), emerged as the most promising therapeutic candidates. CX3CR1, previously identified as the most differentially expressed gene between survivors and non-survivors of septic shock (65, 66), has been linked to impaired ex vivo monocyte function (66) and recognized as an independent biomarker for mortality prediction in critically ill patients (67). FPR1, a chemoattractant GPCR predominantly expressed in neutrophils, macrophages, and monocytes, plays a critical role in immune modulation and inflammation (68), positioning it as a compelling therapeutic target in sepsis (69, 70). Similarly, C3AR1, a marker of CD16^+^ monocytes with a macrophage-like morphology and heightened cytokine production (71), is significantly upregulated and identified as a potential therapeutic target in sepsis (72).

More importantly, we identified interactions between 335 human gut microbial metabolites (33) and sepGIKs using molecular docking-based virtual screening, offering insights into host-microbiome interactions. Overall, sepGIKs were primarily regulated by nucleosides, nucleotides, and their analogues, as well as phenylpropanoids and polyketides, with mean binding affinities of –6.82 kcal/mol and –6.25 kcal/mol, respectively (**Supplementary Figure S5**). These findings align with prior reports highlighting the pharmacological potential of phenylpropanoids and polyketides, known for their anticarcinogenic, neuroprotective, and anti-inflammatory properties both *in vitro* and *in vivo* by targeting cell receptors, enzymes, and related substructures (73). Nucleosides and nucleotides are critical therapeutic agents for various conditions, including cancer, viral infections, and immunosuppression (74).

Tier 1 sepGIKs included 50 metabolite-sepGIK pairs, linking 25 sepGIKs to 31 human gut microbial metabolites (**Figure 4A**; **Supplementary Table S14**). KCNJ15 and FPR1 emerged as key sepGIKs, supported by both genetic and multi-omics evidence (**Figure 4B**). KCNJ15, an inward-rectifying potassium ion channel (75), plays a crucial role in bacterial clearance during infection (76). FPR1 and KCNJ15 interact with N-acetyltryptophan and esculin, respectively (**Figure 4A**). Esculin has demonstrated efficacy in murine models of sepsis (77, 78). Our study also identified MCHR1 (melanin-concentrating hormone receptor 1) as another Tier 1 sepGIK, with esculin emerging as its 1^st^ candidate metabolite. Moreover, IGF1R and MCOLN1, supported by MR_eQTL_, SMR, and colocalization evidence (**Figure 4B**), exhibited strong binding affinities for chlorogenic acid, which is widely recognized for its anti-inflammatory effects in sepsis (79). Here, we identified the 1^st^ candidate metabolite for both DSTYK and KISS1R (kisspeptin 1 receptor) as chlorogenic acid (**Supplementary Table S14**). DSTYK, a member of the RIPK5 kinase family, plays a critical role in mediating cellular stress responses induced by infection, inflammation, or tissue injury (80). Kisspeptin is the natural ligand for KISS1R (also known as GPR54), which has been implicated in various physiological and pathological contexts and was recently found to be significantly elevated in septic patients (81). Activated KISS1R can prevent inflammation by inhibiting NF-κB signaling in nonalcoholic fatty liver disease (82). Interestingly, P2RY14 (G protein-coupled purinergic P2Y receptor), supported by both genetic and multi-omics evidence (**Figure 4B**), displayed the highest number of interactions with gut microbial metabolites (19/50, 38%; **Figure 4A**; **Supplementary Table S14**). P2Y receptors play a pivotal role in inflammation and are involved in various cellular processes, including efferocytosis, phagocytosis, chemotaxis, degranulation, pathogen elimination, cytokine production, and platelet aggregation (83). CALCRL (calcitonin receptor-like receptor), a Tier 1 GPCR supported by robust MR and SMR evidence (**Figure 4B**), plays a crucial role in maintaining vascular integrity and modulating inflammatory responses. Notably, CALCRL downregulation in sepsis disrupts its interaction with RAMP2/3 (receptor activity-modifying proteins), thereby impairing adrenomedullin clearance and compromising its protective anti-inflammatory functions (84–86). CALCRL was predicted to potentially bind with lumichrome (**Figure 4A**). Likewise, serine/threonine kinases MAP2K5 and MAP3K1, both classified as Tier 1 targets, are integral components of the MAPK signaling cascade, orchestrating oxidative stress responses, inflammatory mediator release, and apoptosis during sepsis (87, 88). The 1^st^ candidate metabolite for MAP2K5 is lumichrome and for MAP3K1 is S-adenosylhomocysteine (SAH). We also identified phenylacetylglutamine (PAGln), a microbiota-derived metabolite of phenylalanine, as the 1^st^ metabolite for Tier 1 target NRBP2 (nuclear receptor binding protein 2) (**Figure 4A**). A recent metabolomics study highlighted PAGln as a characteristic biomarker for sepsis diagnosis (89). Tier 2 sepGIKs included 89 sepGIKs (399 pairs), such as FFAR2 (free fatty acid receptor 2), CCR7 (C-C chemokine receptor type 7), HK3 (hexokinase 3), and IRAK3 (interleukin-1 receptor-associated kinase 3), which were differentially expressed in at least 14 sepsis datasets (**Supplementary Table S11**). Notably, chlorogenic acid (79) was identified as their top candidate metabolite (**Supplementary Table S14**). Collectively, these findings provide a comprehensive network linking human gut microbial metabolites with sepGIKs.

We next prioritized causal gut microbial metabolites linked to sepsis using MR analysis. In total, 18 gut microbial metabolites with high abundance in human gut microbiota genera (33) were found to be associated with sepsis (**Supplementary Figure S6**; **Supplementary Table S15B**). Some genera, such as *Clostridioides* spp (90)., *Bifidobacterium* spp (91)., *Bacteroides* spp (92)., *Akkermansia* spp (93)., *Blautia* spp (94)., and others, have been previously reported to be associated with sepsis. Notably, 5-oxoproline, produced by *Parabacteroides johnsonii*, exhibited the strongest association with an increased risk of sepsis-related mortality (β_IVW_ = 0.249, *p* = 1.04×10^-5^). Accumulation of 5-oxoproline has been shown to induce inflammation and impair antioxidant defenses (95, 96), and it has been notably enriched in *Pseudomonas aeruginosa*-related sepsis (97). 5-oxoproline has recently proven effective in sepsis diagnosis and risk stratification (98). Additionally, *Parabacteroides johnsonii* has been found to be enriched in patients with severe acute pancreatitis (99). Collectively, these findings support the existence of likely causal relationships between gut microbial metabolites and sepsis pathogenesis. We then explored the potential interactions between sepsis-related gut microbial metabolites and sepGIKs. Notably, the strongest binding interaction was observed between PFKFB2 and SAH (affinity = -9.0 kcal/mol; **Supplementary Table S13**). Elevated levels of SAH have been linked to more severe septic illness and identified as an independent predictor of sepsis progression, mortality, and a marker of hypoxia-induced organ dysfunction (100, 101). PFKFB2, which is significantly upregulated in sepsis, has also been implicated in promoting neutrophil inflammatory activation (59). Furthermore, we identified N-acetylputrescine, a potential sepsis biomarker (102, 103) with high abundance in *Proteus* (log_2_FC = 9.62; **Supplementary Table S4**), as a candidate binding metabolite for the Tier 1 sepGIK LGR6 (**Figure 4B**). Targeting LGR6 has potential to resolve inflammation, enhance macrophage phagocytosis, and promote tissue repair in sepsis (104, 105).

Sepsis initiates a complex interplay between pro-inflammatory and anti-inflammatory responses, resulting in a significant disruption of immune homeostasis (106). Hyperinflammation, a hallmark of sepsis, contributes to organ damage, while the subsequent immunosuppression exacerbates the risk of secondary infections, rehospitalization, and long-term mortality (107, 108). In this study, we found that CX3CR1 and MCOLN1 were downregulated in both low-activity T cells and low-HLA-DR monocytes (**Figure 6B**). Consistent with our findings, previous studies have shown that reduced CX3CR1 expression on circulating monocytes is a hallmark of sepsis-induced immunosuppression (66). Moreover, MCOLN1-mediated lysosomal exocytosis exacerbates lipopolysaccharide (LPS)-induced secretion of SQSTM1, an autophagy receptor, which has been implicated in immune dysfunction and organ injury in sepsis patients (109). As shown in **Figure 6C**, MCOLN1 was shown to interact with chlorogenic acid, a compound with anti-inflammatory properties (79). Furthermore, CX3CR1 exhibited the strongest binding affinity for lumichrome, a compound known to confer protection against ischemia-reperfusion injury in myocardial tissue (110). PTK2B was found to be downregulated in both low-activity T cells and B cells (**Figure 6B**). This finding aligns with previous studies indicating that PTK2B plays an essential role in immune-mediated inflammatory diseases, modulating the development and regulation of T and B cell responses (111, 112). PTK2B exhibited the strongest binding affinity for esculin (**Figure 6C**), a compound with therapeutic potential in sepsis (77, 78). PTGIR expression was significantly altered in monocytes (**Figure 6B**). Previous studies have shown that in LPS-stimulated THP-1 human monocytes, PTGIR antagonists suppress the expression of proinflammatory cytokines TNF-α and MIP-1α (113, 114), as well as the calprotectin subunits S100A8/A9 (115). Excessive S100A8/A9 production drives a positive feedback loop, worsening sepsis-induced hyperinflammation and increasing mortality (116, 117). Interestingly, PTGIR and esculin were identified as each other’s 1^st^ compounds (**Figure 6C**; **Supplementary Table S14**), underscoring their synergistic role in modulating the immune response in sepsis (77, 78). Furthermore, four Tier 1 sepGIKs, including ZAP70, FPR1, NME4, and C3AR1, were differentially expressed in T cells (**Figure 6B**). ZAP70, a critical tyrosine kinase involved in T cell activation following T cell receptor (TCR) engagement (118), has been identified as a potential biomarker for sepsis in multiple bioinformatics studies (119–121). In our study, ZAP70 was differentially expressed in nine sepsis datasets (**Supplementary Table S11**) and validated through genetic-based MR_eQTL_ and SMR analyses (**Figure 4B**). NME4 regulates CD8^+^ T cell infiltration into the tumor microenvironment (122). Both ZAP70 and NME4 showed the strongest binding affinity for lumichrome (**Figure 6C**), highlighting the potential relevance of this metabolite in sepsis-induced immunosuppression. BLK, a kinase involved in B cell subset development and B cell receptor (BCR) activation, was differentially expressed in B cells in the context of sepsis (**Figure 6B**) (123). In this study, BLK exhibited differential expression in six independent sepsis datasets (**Supplementary Table S11**) and causal association in four distinct sepsis outcomes (FDR_IVW_ < 0.05; **Figure 2**). Notably, its top candidate metabolite is chlorogenic acid (**Figure 6C**) (79). In summary, these findings provide a comprehensive overview of potential gut microbial metabolite-sepGIK interactions that contribute to immunosuppression during sepsis. The identification of these associations offers promising candidates for future experimental validation, which could yield novel therapeutic strategies for sepsis-associated immune dysfunction.

We finally prioritized human gut metabolites derived from several specific sepsis-protective microbiota. For instance, *L. rhamnosus* has been shown to mitigate sepsis-induced cognitive impairment (124) and intestinal injury (125), while *L. plantarum* reduces endotoxemia triggered by alcohol and high-fat diets (126, 127). Similarly, *B. infantis* lowers sepsis risk in human-milk-fed infants (128), and *B. pseudocatenulatum* demonstrates therapeutic potential in severe acute pancreatitis (129). By inspecting metabolite profiles and their abundances (**Supplementary Table S4**), we found that D-glucose 6-phosphate displayed a remarkable accumulation in *B. pseudocatenulatum* (log_2_FC = 12.66), with its 1^st^ sepGIK identified as pyruvate kinase (PKM; **Supplementary Figure S7**), a key regulatory enzyme in glycolysis and a promising target for sepsis intervention (10). Beyond these classical probiotic genera, *Akkermansia muciniphila* (*A. muciniphila*) has emerged as a next-generation probiotic with compelling therapeutic potential across various diseases, including sepsis (57, 58). Notably, *A. muciniphila* mitigates sepsis-induced acute kidney injury (130), while a purified membrane protein from this species confers protection against acute lung injury (131). Additionally, the tripeptide RKH, derived from *A. muciniphila*, functions as a novel TLR4 antagonist, offering significant protective effects against lethal sepsis (132). Strikingly, ILA was consistently enriched across all five sepsis-protective microbial species (log_2_FC ≥ 2; **Figure 7A**), suggesting a protective role in modulating host responses to sepsis. As shown in **Supplementary Figure S7**, PFKFB2 (59) is the 1^st^ sepGIK for ILA. Importantly, MD simulation and MST analysis further establish PFKFB2 as a potential molecular target through which ILA exerts its pharmacological effects. Microbiota-derived ILA has been shown to alleviate both intestinal (133, 134) and neuroinflammation (135, 136); however, direct evidence linking ILA to sepsis remains limited. To address this, we employed the CLP model, a well-established and clinically relevant experimental model of sepsis. As anticipated, ILA administration significantly enhanced survival and protected against multi-organ dysfunction in CLP-injured mice. Given that multi-organ dysfunction in sepsis is primarily driven by a hyperinflammatory cytokine storm (137), we examined serum levels of TNF-α, IL-6, and IL-1β and found that ILA effectively mitigated systemic inflammatory response. Collectively, our findings identify ILA as a potential therapeutic agent in sepsis, conferring protection by dampening systemic inflammation and preserving organ integrity.

In conclusion, our study represents a paradigm shift by integrating multi-omics profiling, genetics-driven computational analysis, and structure-based virtual screening to systematically elucidate the interplay between human gut microbiota-derived metabolites and the druggable GIKome in sepsis. This systems-level investigation unveils previously unrecognized therapeutic targets, offering new avenues for microbiota-based precision interventions in critical care medicine. Several limitations of our study should be acknowledged. First, the datasets analyzed predominantly comprised individuals of European ancestry, which may limit the generalizability of our findings to other ethnic groups. Future research should prioritize diverse populations to enhance the broader applicability of these results. Second, our eQTL analyses were based on blood-derived transcriptomic data rather than tissues directly implicated in sepsis-induced MODS, which may constrain the physiological relevance of our findings. However, blood-based biomarkers remain highly attractive for clinical translation due to their feasibility in storage, detection, and therapeutic targeting. Third, although our current study focused primarily on the gut bacterial microbiota, we acknowledge that the intestinal microbial ecosystem is far more complex and includes other key components such as fungi, viruses, archaea, and their metabolites. For example, increasing evidence highlights the critical roles of gut fungi, notably Candida and Saccharomyces spp., in shaping bacterial community structure, modulating host immunity, and contributing to disease pathogenesis through cross-kingdom interactions (138). These complex inter-kingdom relationships may have influenced the outcomes observed in our study. However, due to the limitations of the available dataset (e.g., 16S rRNA-based sequencing), we were unable to capture non-bacterial taxa. Future studies integrating multi-omics approaches (e.g., ITS sequencing, metagenomics) are warranted to provide a more comprehensive understanding of the gut microbial ecosystem and its role in host health and disease. Another limitation is the absence of protein-level validation for certain preliminary findings, as many pQTLs corresponding to key genes lacked SNPs after filtering. Additionally, the reliance on AlphaFold2 for structural modeling introduces potential biases, as the model tends to predict either the active or inactive conformation of GIKs, leaving the agonistic or antagonistic effects of metabolites on these targets uncertain. Lastly, further research is needed to elucidate the mechanisms of action and metabolism of these metabiotics, paving the way for longitudinal studies and clinical trials to validate their therapeutic potential. Especially, while we observed a link between ILA treatment and reduced glycolytic activity, direct genetic evidence to confirm the involvement of PFKFB2 is currently lacking. Future studies using genetic manipulation of PFKFB2 will be critical to firmly establish its causal role in mediating the effects of ILA on glycolysis.

## Data Availability

The datasets presented in this study can be found in online repositories. The names of the repository/repositories and accession number(s) can be found in the article/**Supplementary Material**.
