# Supplementary material for: Comprehensive characterization of multi-omics landscapes between gut microbial metabolites and the druggable genome in sepsis
Source: Front Immunol. 2025 Jul 21;16:1597676. doi: 10.3389/fimmu.2025.1597676 (PMC12318984; doi:10.3389/fimmu.2025.1597676)
Supplement: Supplementary file 1 [file DataSheet1.pdf]

Supplementary Figures:

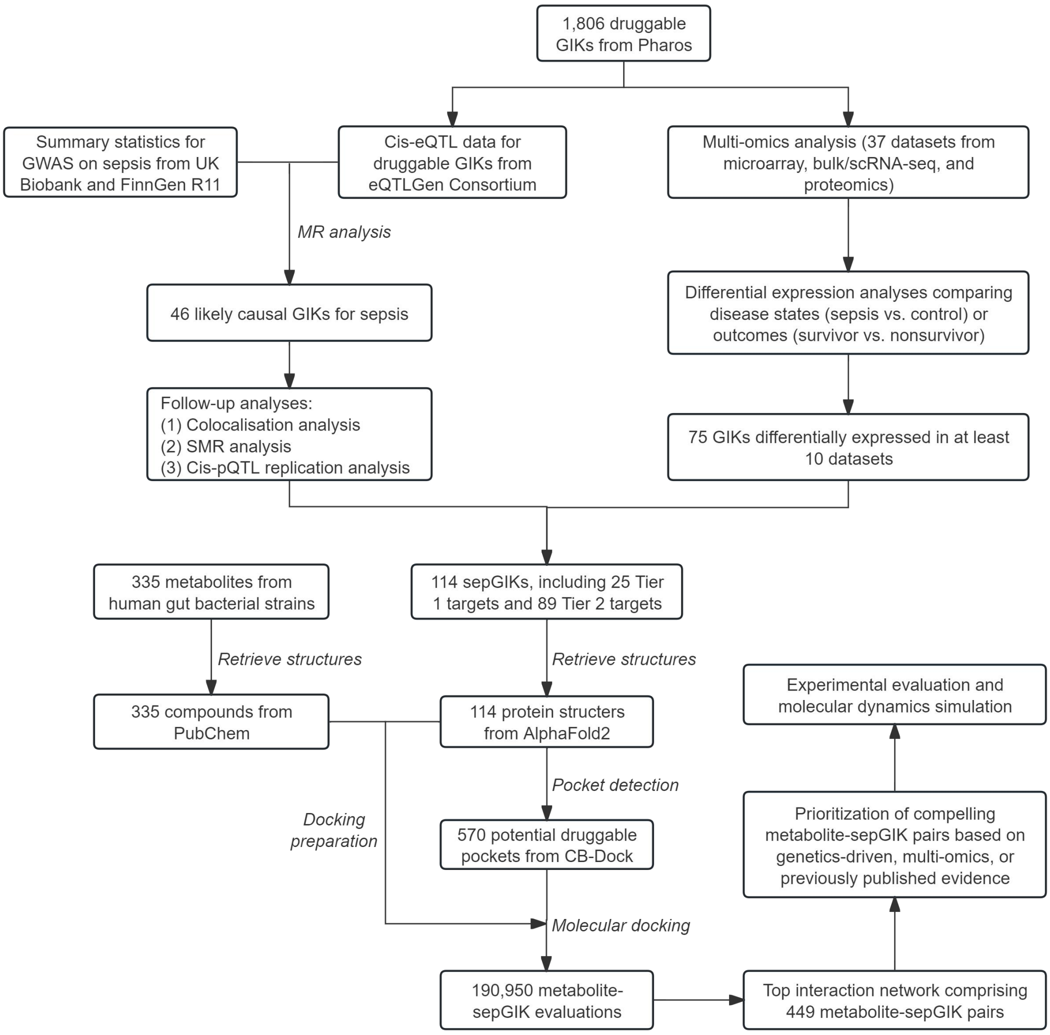

Figure S1. Comprehensive schematic of the workflow in our study. A Tier 1 target is defined as one supported by at least two genetically derived pieces of evidence or by one such piece of evidence coupled with differential expression in at least five sepsis transcriptomic or proteomic datasets, or by differential expression across 15 or more datasets. A Tier 2 target is defined as one supported by at least one genetic evidence or differential expression in at least 10 datasets. A curated list of 335 metabolites derived from 158 human gut bacterial strains was compiled from recent in vitro studies conducted by Han et al. The 449 metabolite-sepGIK interaction pairs was constructed by integrating docking-derived binding affinities, specifically retaining the top-ranked sepGIK for each metabolite and the top metabolite for each sepGIK. sepGIKs, sepsis-related G-protein-coupled receptors (GPCRs) , ion channels (ICs), and kinases, determined by genetics-driven and multi-omics evidence; GWAS, genome-wide association study; eQTL, expression quantitative trait locus; pQTL, protein quantitative trait locus; SMR, summary data-based Mendelian randomization.

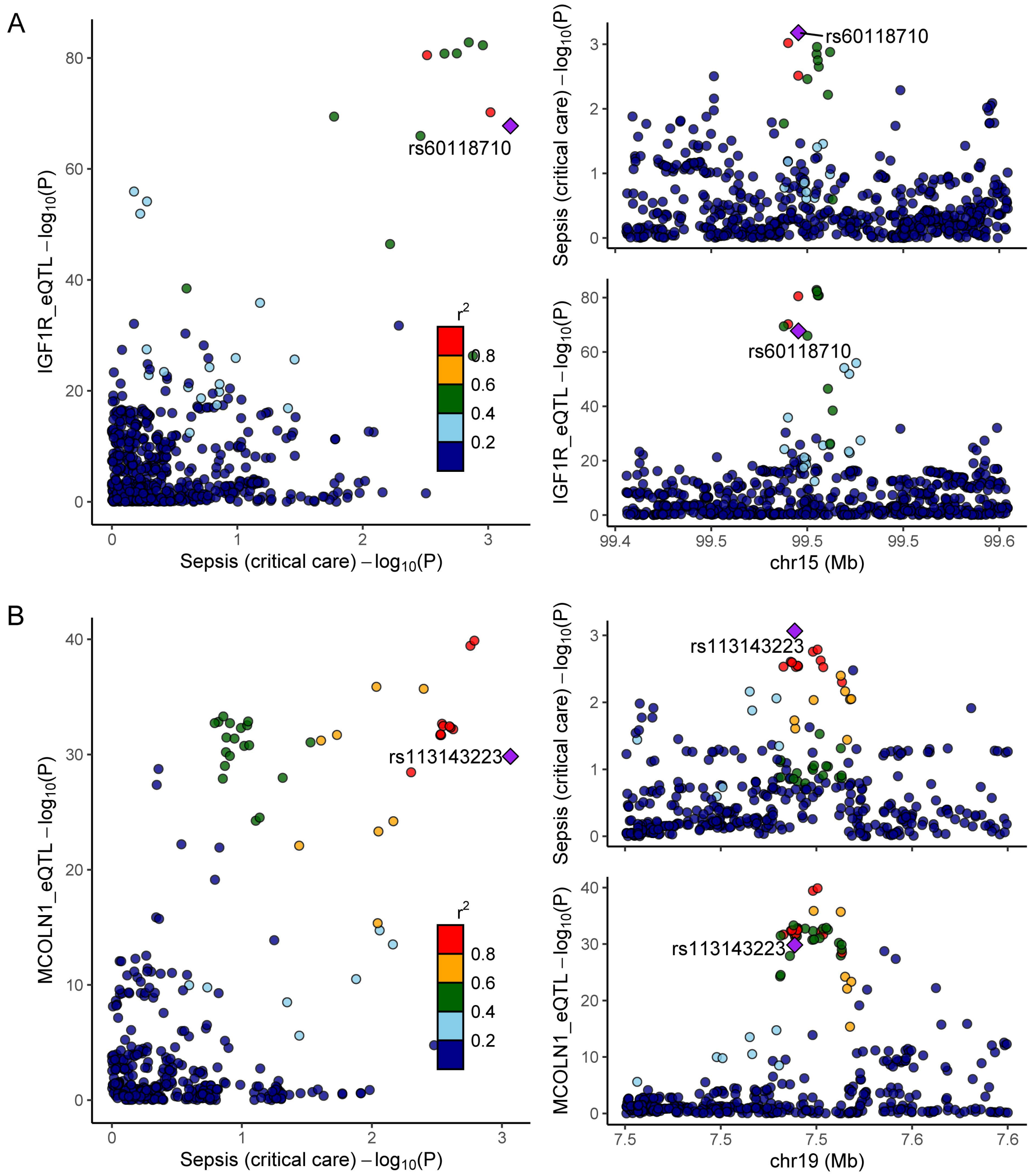

Figure S2. The results of colocalization analysis. (A) The association of IGF1R with sepsis in critical care. (B) The association of MCOLN1 with sepsis in critical care.

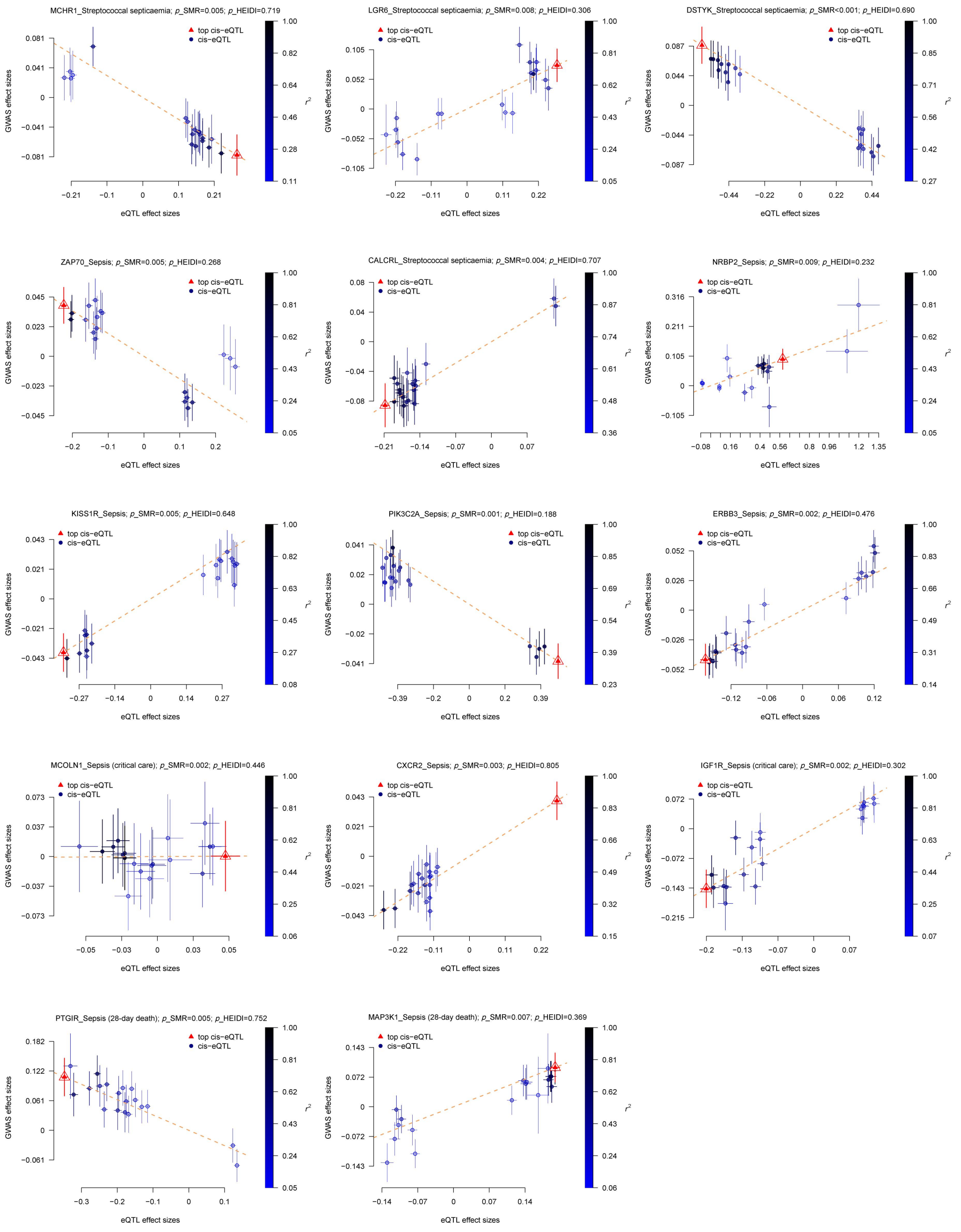

Figure S3. The summary-data-based Mendelian randomization (SMR) effect plots of significant GIKs with sepsis outcomes. The horizontal axis represents the effect sizes of SNPs on GKIs, while the vertical axis represents the effect sizes of SNPs on sepsis outcomes.

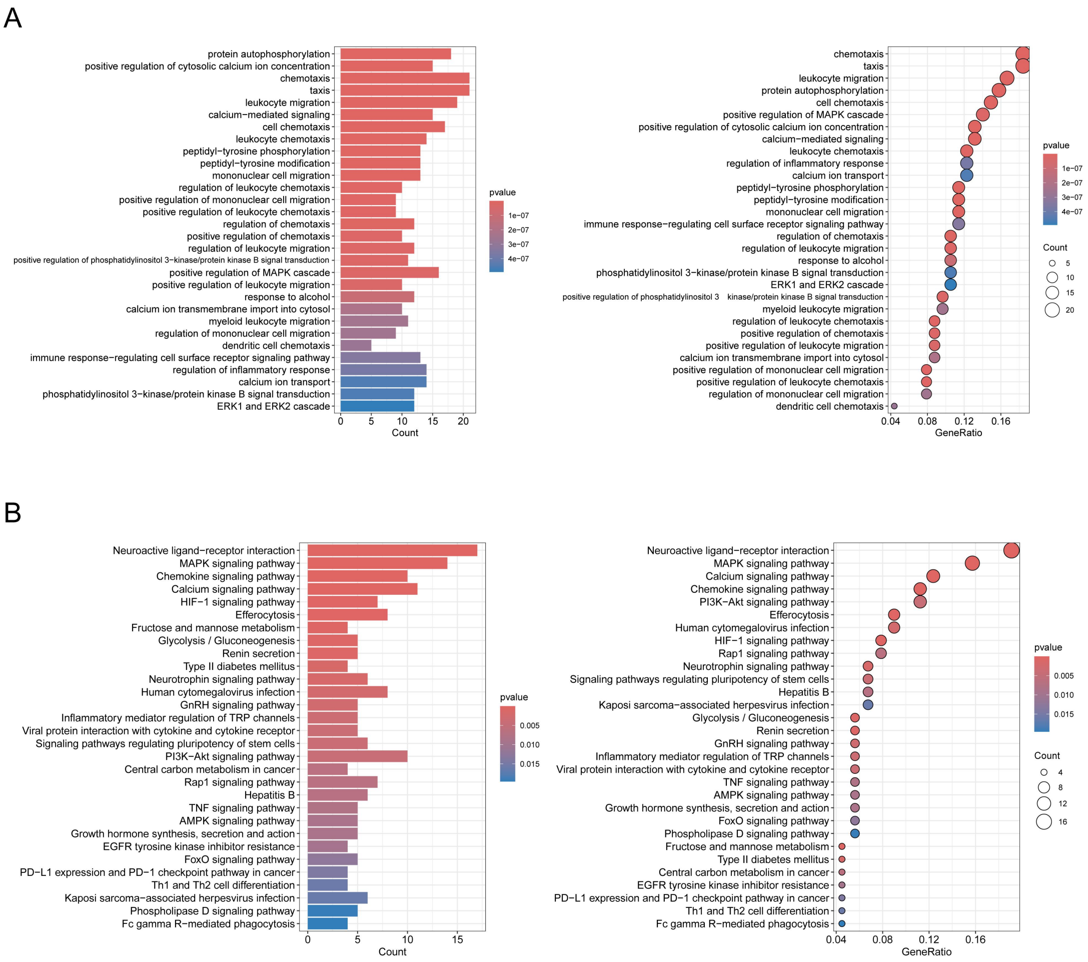

Figure S4. GO and KEGG enrichment analyses for 114 sepGIKs. (A) GO results. (B) KEGG results.

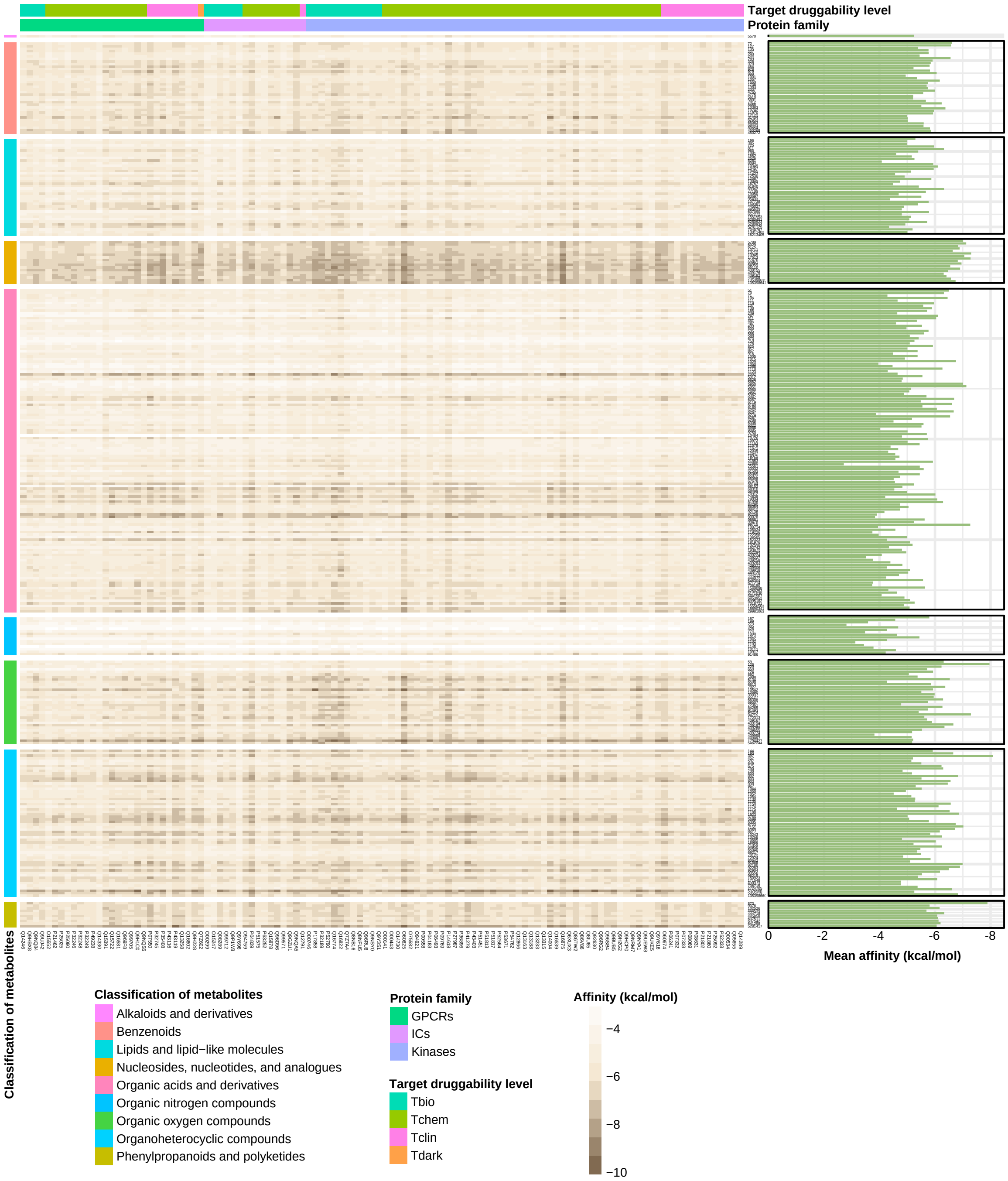

Figure S5. Docking score profiles of metabolite-sepGIK interactions. A heatmap depicting associations between 114 sepGIKs (by column) and 335 human gut microbial metabolites (by row). The classes of sepGIK and metabolite are displayed aside with different color.

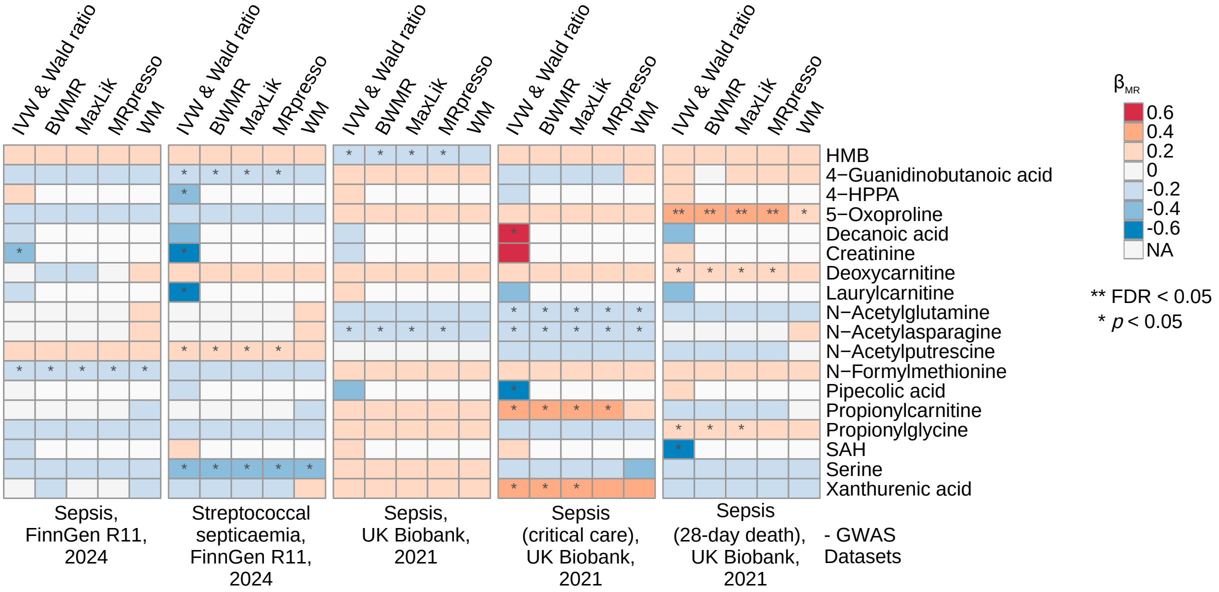

Figure S6. Prioritization of human gut microbial metabolites through MR analysis. In total, five sepsis GWAS datasets from the UK Biobank and FinnGen were used. The GWAS dataset for blood metabolite levels was obtained through the research by Chen et al. β > 0 indicates that increased expression of a metabolites is associated with a higher likelihood of sepsis.

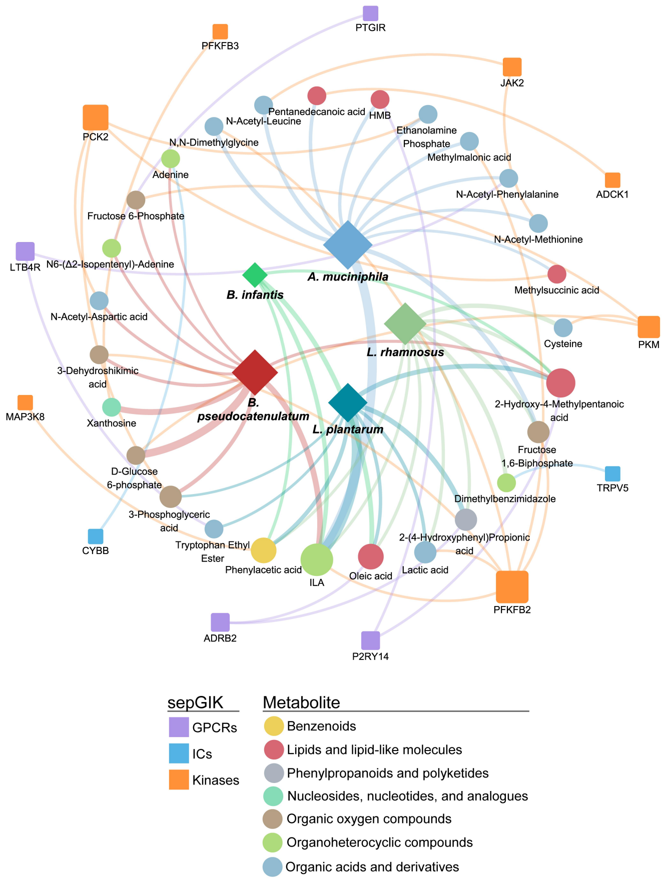

Figure S7. Interaction network between gut metabolites (from *L. rhamnosus*, *L. plantarum*, *B. pseudocatenulatum*, *B. infantis*, and *A. muciniphila*) and sepGIKs. Only the metabolite with higher abundance (|$\log_2$FC| ⩾ 2, compared to germ-free controls) in these five sepsis-protective bacteria genera are shown. A docking score (edge) connecting the 1st metabolite of sepGIKs or the 1st sepGIK of metabolites. The color of shape and lines are based on bacteria genus types. The line width indicates the abundance of metabolite in the bacteria. Metabolite and sepGIK are depicted as circle and rectangle nodes, respectively. Protein family class of sepGIKs and chemical class of metabolites are indicated with different colors. The network was visualized using Cytoscape.

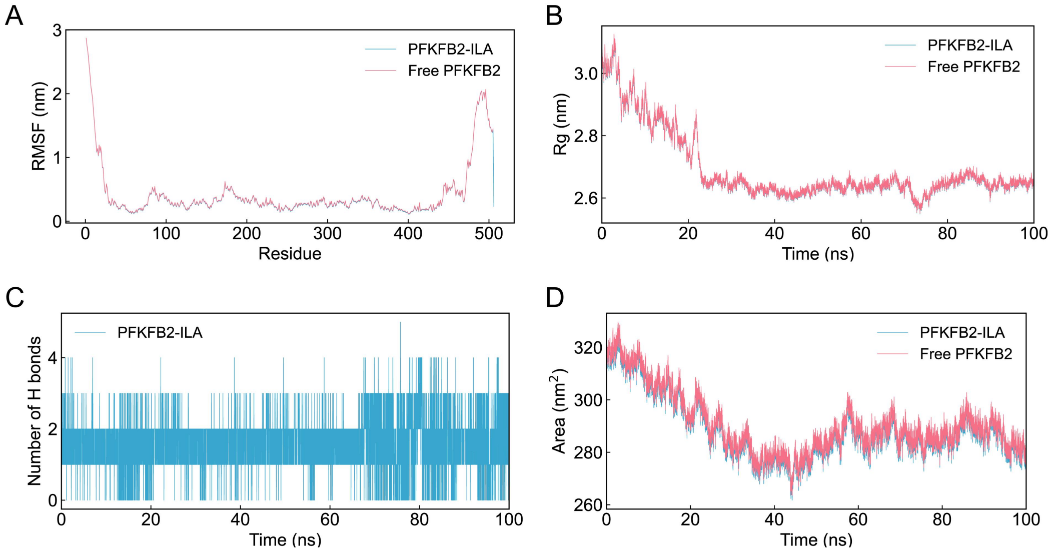

Figure S8. Post-analysis of molecular dynamics simulation for PFKFB2-ILA complex. (A) RMSF (Root Mean Square Fluctuation) analysis, highlighting residue flexibility across the complexes. (B) Radius of gyration (Rg) analysis, indicating molecular compactness. (C) Hydrogen bond count analysis, detailing the number of hydrogen bonds formed over time. (D) SASA (Solvent Accessible Surface Area) analysis, showing solvent exposure levels.

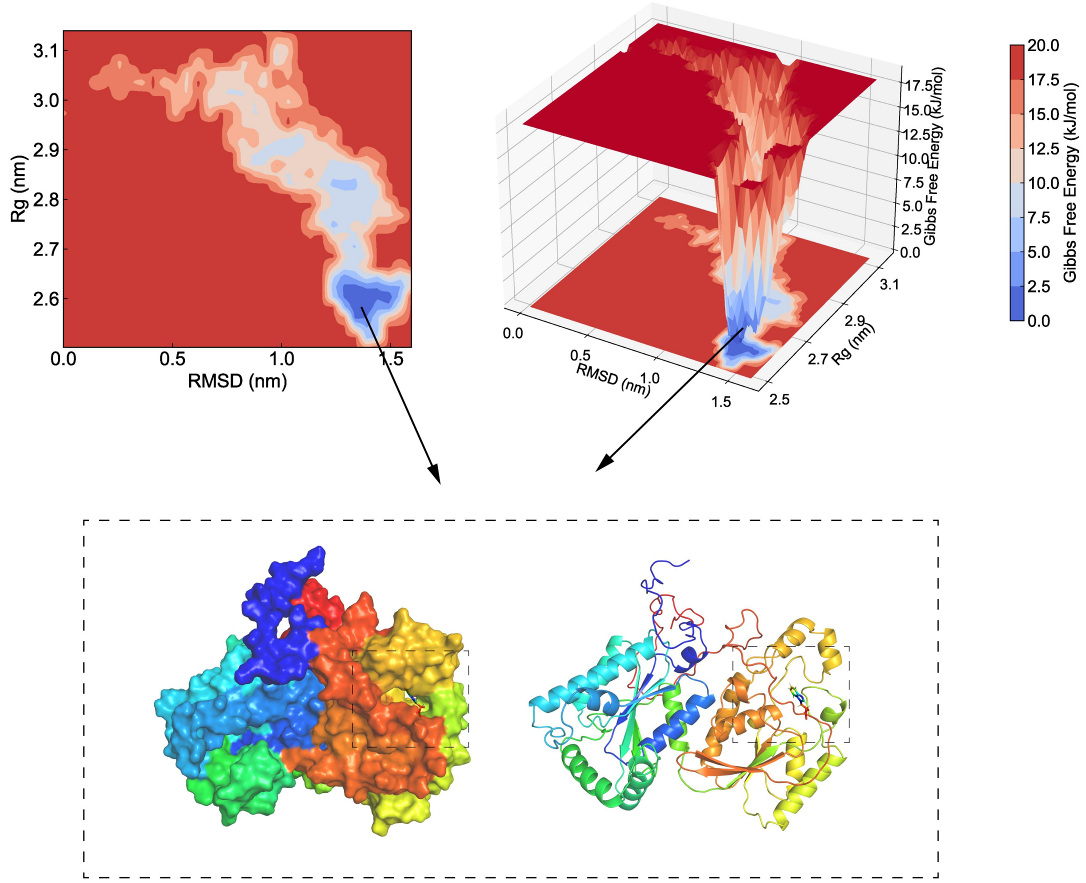

Figure S9. Gibbs Energy Landscape and lowest energy conformation of PFKFB2-ILA complex, with darker regions indicating lower binding energies.

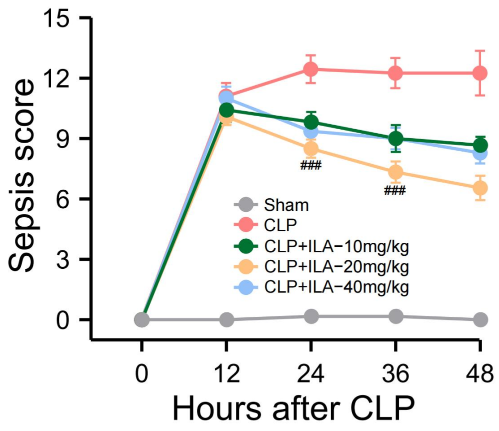

Figure S10. Effects of ILA pretreatment on murine sepsis score (MMS) in CLP-induced mice. Sepsis severity was assessed according to the MSS obtained by checking variables, including appearance, level of consciousness, activity, response to stimulus, eyes, respiration rate, and respiration quality, as established by Shrum et. al. $^{###}P < 0.001$ versus CLP group.

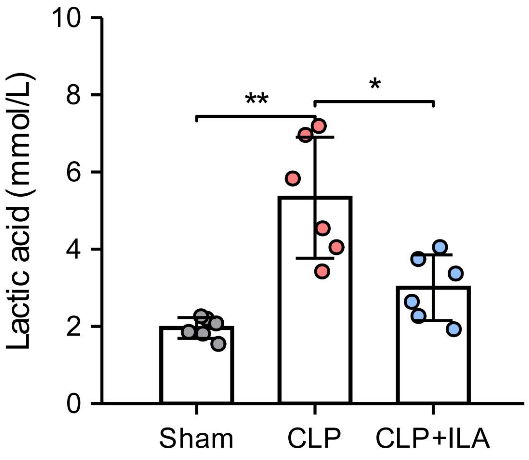

Figure S11. Lactic acid levels in mouse blood were detected. *$p < 0.05$, **$p < 0.01$, ***$p < 0.001$.

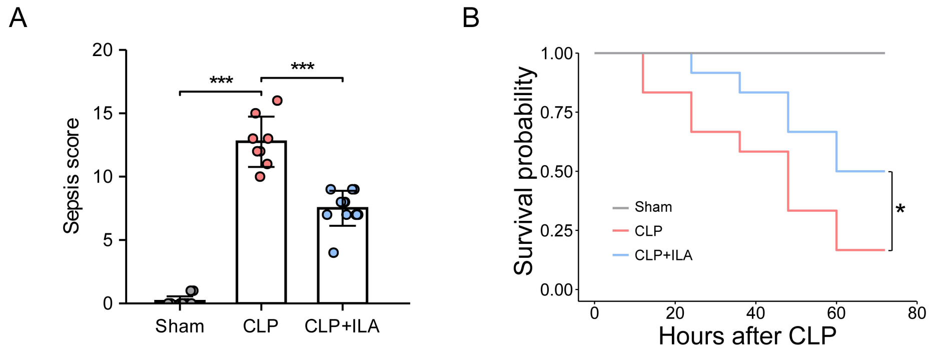

Figure S12. Effects of post-CLP administration of ILA on MSS score and survival in mice. ILA was administered intraperitoneally at a dose of 20 mg/kg following CLP. The administration was repeated every 12 hours for three consecutive days. (A) MSS was assessed 24 hours after CLP. (B) The Kaplan-Meier survival curves assessed for up to 72 hours. Each line represents the survival of mice in a group; 12 mice were in each group. *p < 0.05, **p < 0.01, ***p < 0.001.
